# Dietary ω3 Fatty Acids and Phytosterols in the Modulation of the HDL Lipidome: A Longitudinal Crossover Clinical Study

**DOI:** 10.3390/nu15163637

**Published:** 2023-08-18

**Authors:** Teresa Padro, Anallely López-Yerena, Antonio Pérez, Gemma Vilahur, Lina Badimon

**Affiliations:** 1 Cardiovascular Program-ICCC, Institut d’Investigació Biomèdica Sant Pau (IIB SANT PAU), 08041 Barcelona, Spain; naye.yerena@gmail.com (A.L.-Y.); gvilahur@santpau.cat (G.V.); lbadimon@santpau.cat (L.B.); 2 Centro de Investigación Biomédica en Red Cardiovascular CIBERCV), Instituto de Salud Carlos III, 28029 Madrid, Spain; 3 Servicio de Endocrinología y Nutrición, Hospital de la Santa Creu i Sant Pau, IIB Sant Pau, Universitat Autònoma de Barcelona, 08041 Barcelona, Spain; aperez@santpau.cat; 4 CIBER de Diabetes y Enfermedades Metabólicas Asociadas (CIBERDEM), 08041 Barcelona, Spain; 5 Cardiovascular Research Chair, Universitat Autònoma de Barcelona (UAB), 08193 Barcelona, Spain

**Keywords:** lipids, high-density lipoproteins, DHA, EPA, omega-3 fatty acids, sex

## Abstract

High-density lipoproteins (HDLs) are complex particles composed of a wide range of lipids, proteins, hormones and vitamins that confer to the HDL particles multiple cardiovascular protective properties, mainly against the development of atherosclerosis. Among other factors, the HDL lipidome is affected by diet. We hypothesized that diet supplementation with ω3 (docosahexaenoic acid: DHA and eicosapentaenoic acid: EPA) and phytosterols (PhyS) would improve the HDL lipid profile. Overweight subjects (*n* = 20) were enrolled in a two-arm longitudinal crossover study. Milk (250 mL/day), supplemented with either ω3 (EPA + DHA, 375 mg) or PhyS (1.6 g), was administered to the volunteers over two consecutive 28-day intervention periods, followed by HDL lipidomic analysis. The comprehensive lipid pattern revealed that the HDL lipidome is diet-dependent. ω3-milk supplementation produced more changes than PhyS, mainly in cholesteryl esters (CEs). After ω3-milk intake, levels of DHA and EPA within phosphatylcholines, triglycerides and CE lipids in HDLs increased (*p* < 0.05). The correlation between lipid species showed that lipid changes occur in a coordinated manner. Finally, our analysis revealed that the HDL lipidome is also sex-dependent. The HDL lipidome is affected by diet and sex, and the 4 weeks of ω3 supplementation induced HDL enrichment with EPA and DHA.

## 1. Introduction

High-density lipoprotein (HDL) particles have been demonstrated to possess various antiatherogenic characteristics, encompassing anti-inflammatory, antioxidant, antiapoptotic, antithrombotic and vasodilatory effects [1]. However, the ability to promote cholesterol efflux from macrophages, which is part of the reverse cholesterol transport pathway, is undoubtedly one of the most important postulated antiatherogenic properties of HDLs [2]. HDLs are complex particles composed of a wide range of lipids, proteins, hormones, vitamins and miRNAs that confer on HDL particles multiple cardiovascular protective properties, mainly related to the development of atherosclerosis [3]. Regarding the HDL lipidome, it is predominantly made up of phospholipids, particularly phosphatidylcholine (PC) and sphingomyelin (SM), accounting for 40–60% of the total lipid fraction. In addition, HDLs contain cholesteryl esters (CEs; 30–40%), triglycerides (TAGs; 5–12%) and free cholesterol (5–10%) [4]. Plasma lipoproteins undergo continuous lipid exchange, involving constituents of the lipoprotein surface monolayer, specifically phospholipids and unesterified cholesterol and those belonging to the core lipids, TAGs and CEs [5]. The exchange of lipids with cells and other lipoproteins is conceivably important for the maintenance of HDL structure, stability and function, including metabolism, reverse cholesterol transport or the activity of related enzymes [6]. In view of the foregoing, it is not surprising that the detailed profiling of lipoprotein fractions and modification in their molecular lipid profiles may help identify novel biomarkers and signaling effectors in lipid metabolism and cardiovascular health [6].

It is now well evidenced that the HDL lipidome is significantly altered in pathological conditions like dyslipidemia, hypertension, coronary artery disease [7], type 2 diabetes mellitus [8] and obesity [9], among other comorbid and lifestyle conditions. Indeed, the fact that diet can affect the HDL lipid profile has been demonstrated by recent intervention studies. In one such study, it was shown that the macronutrient content of a meal has an acute impact on regulating the composition of HDLs in the postprandial state, being more affected by the intake of a diet rich in saturated fats than one rich in carbohydrates [10]. In another recent study, it was mainly demonstrated that 3 weeks of virgin olive oil consumption have an impact on TAG species [11]. In addition, 4-day dietary intervention with a fast-food diet or with the Mediterranean diet differentially changed HDL lipidomic composition, demonstrating that certain HDL lipids could be useful markers of short-term dietary intake (PE, PC and CE); alternatively, other HDL lipids (SM and ceramides, Cer) are not as responsive to short-term dietary change [12]. Finally, in another study it was demonstrated that daily egg consumption for 12 weeks promotes an increase in CEs and a reduction in TAGs in the HDL and changes in its functions beyond increasing plasma HDL in patients with metabolic syndrome [13]. However, to our knowledge, very few studies have attempted to discern the effects of natural dietary compound supplements (ω3 fatty acids and phytosterols (PhyS)) on the HDL lipidome. A very recent study demonstrated that DHA and EPA supplementation induced enrichment with both lipids to the detriment of some *n*-6 fatty acids [14]; however, a detailed lipidomic evaluation was not carried out.

We previously reported that daily intake of milk products containing ω3 (docosahexanoic acid: DHA and eicosapentaenoic acid: EPA) affects the composition of the HDL proteome, with a significant increase in key proteins involved in HDL metabolism [15]. Using an LDL mass spectrometry approach, we have also evidenced that dairy (milk) products fortified with ω3 or PhyS induce changes in the LDL lipidome beyond their lowering effect on plasma TGL and cholesterol levels [16]. Here, we focused on a similar high-throughput discovery approach to gain better understanding of the modulatory effects of sustained intake of low-fat milk supplemented with ω3 fatty acids or with PhyS on the HDL lipidome that might relate to a decrease in atherosclerotic risk in overweight individuals.

## 2. Materials and Methods

### 2.1. Study Design

**Figure 1:**
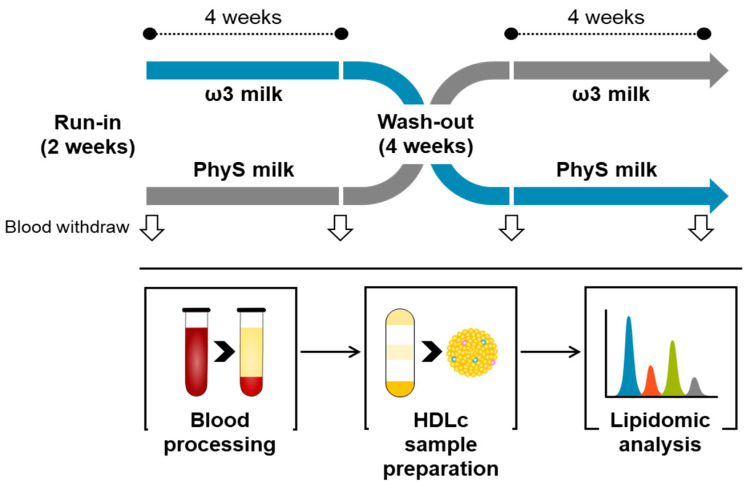
Flow diagram describing the study design.

The current study is an exploratory sub-study of a double-blind randomized two-arm longitudinal crossover intervention trial [clinical trial: ISRCTN78753338] [16]. Briefly, in a double-blind randomized two-arm longitudinal crossover trial, the volunteers were submitted to two 28-day intervention periods with dairy products enriched in PhyS or ω3 fatty acids, separated by a 4-week wash-out period (Figure 1). The experimental design and the characteristics of the volunteers have previously been described [16]. Prior to the initiation of the intervention, individuals had a 2-week preliminary period (run-in). Participants (*n* = 20) were instructed to consume 250 mL per day of ω3-supplemented milk (131.25 mg EPA + 243.75 mg DHA/250 mL of milk; total ω3 supplement 375 mg/day) or PhyS-supplemented milk (1.6 g of plant sterols/250 mL of milk; total supplement 1.6 g/day) during the intervention periods. The provided milk was consumed (total 250 mL/day) according to the normal habits of each participant and replaced the dairy products they consumed on a regular basis. During the run-in and wash-out periods, volunteers received commercially available plain low-fat milk (free of ω3 and PhyS) possessing identical formulation to that used to prepare the supplemented milks, according to the study procedure indicated above. ω3 milk and PhyS milk were prepared by CAPSA Food (Spain) and managed without product identification. PhyS milk was commercially accessible, whereas ω3 milk was specifically prepared for the purposes of the study. Composition of the intervention products has been described in detail in [16].

Individuals were not considered for inclusion if they indicated chronic illnesses including cancer, overt hyperlipidemia, hypertension, diabetes mellitus or heart, liver or kidney disease and history of CVD. In addition, receiving anti-inflammatory medication or anticoagulant treatment during the study or during the two-week run-in period was also considered as a criterion for exclusion. Additional exclusion criteria included the following: use of lipid-lowering drugs, diuretics, or β-blockers, lactose intolerance, or being in a weight-loss program. Volunteers who ingested a spread enriched with PhyS or fish oil supplements or exhibited a pronounced aversion to dairy products were also disqualified from participation. A thorough physical examination administered by the study’s physician was conducted to verify the participants’ healthy condition.

All study participants were instructed to maintain their usual diets throughout the study period (12 weeks), consistent with stable weight maintenance, and to continue their normal patterns of physical activity. Dietary and physical activity habits and rare changes were recorded by the volunteers in food frequency questionnaires and participants’ diaries. The diaries were delivered at each visit at the end of run-in period and the end of each phase of intervention period. Compliance was monitored through weekly telephone calls and personal interviews and with a written form in each period. The study received approval from the Human Ethical Review Committee of Hospital Santa Creu I Sant Pau (Barcelona), with the reference number 10/2011 and the date of approval being 5 July 2011. All participants gave informed written consent prior to inclusion.

### 2.2. Blood Sampling

Blood samples, obtained following a twelve-hour fasting period, were collected on days 1 and 28 to establish baseline and first-treatment-period endpoints. Similarly, on days 56 and 84, representing baseline and second-treatment-period endpoints, fasting blood samples were obtained, all in accordance with the previously stipulated procedural framework [15,16] (Figure 1). Blood samples for HDL lipidomic studies were collected in EDTA-containing Vacutainer tubes and the separation of plasma was accomplished through centrifugation (3000× *g*, 20 min at 4 °C); the samples were aliquoted and stored at −80 °C until analysis.

### 2.3. HDL Sample Preparation and Purity Control

Lipoprotein fractions were prepared as previously described [17]. The KBr density gradients 1.019–1.063 and 1.063–1.210 were used for LDL and HDL, respectively. Lipoprotein purity was assessed using electrophoresis (2 µL sample) in agarose gels using a commercial assay (SAS-MX Lipo 10 kit; Helena Biosciences, Gateshead, UK), as described by the providers. Additionally, the examination of HDL purity was conducted via analysis of the HDL profile within samples obtained from subjects selected in a random fashion (one subject per ultracentrifugation batch) using chromatography analysis with microgel filtration using a Superose 6 PC 3.2/30 column and an Agilent 1200 HPLC system. Briefly, undiluted HDL sample fraction (10 µL) was loaded in the system and run with a constant flow of 100 µL/min. A comparison was made between the retention time of the HDL fraction (134 min) and that of the LDL fraction (130 min).

### 2.4. Metabolite Extraction

For the glycerolipid (GLP), CE, sphingolipid (SPL) and glycerophospholipid (GPL) profiling, the following method modified from that of Barr et al. [18,19] was used. Seventy-five μg of proteins were taken to obtain a final volume of thirty-five μL in PBS. This volume was mixed with 115 μL of water and 1050 μL of refrigerated chloroform/methanol (2:1 *v*/*v*) in 1.5 mL microtubes. Later, the samples were homogenized and incubated for 30 min at −20 °C. After this time, the samples were homogenized and 1 mL was transferred into another microtube. The samples were mixed with 150 μL of water (pH = 9) and were incubated for 1 h at −20 °C. After centrifugation (16,000× *g*, 15 min at 4 °C), 360 μL of the organic phase was collected and the solvent was removed at 40 °C. The dried extracts were then reconstituted in 100 μL of ACN/isopropanol (1:1 *v*/*v*), mixed for 10 min at room temperature and centrifuged (16,000× *g* for 5 min) and finally transferred to vials for UPLC^®^-MS analysis. Moreover, two distinct quality control (QC) samples were employed for evaluating the quality of the data. The QC samples consist of reference serum specimens that are uniformly allocated across the batches and subjected to extraction and analysis concurrently with the individual samples. The first ones (QC calibration sample) were used to correct the different response factors between and within batches. The second ones (QC validation sample) were used to assess how much data preprocessing procedure improved the data quality. The QC calibration and validation extracts were uniformly interspersed throughout the entire batch run.

### 2.5. Lipidomic Pattern Analysis

The lipid separations were carried out on an AcquityTM UPLC^®^ BEH C18 column (2.1 × 100 mm, i.d., 1.7 µm particle size) (Waters Corporation^®^, Wexford, Ireland). The mobile phase A consisted of 10 mM ammonium acetate in H_2_O:ACN and the mobile phase B consisted of 10 mM ammonium formate in ACN:isopropanol. The elution was performed by means of an increasing linear gradient (*v*/*v*) of B (%, min), as follows: (40, 0); (100, 10); (40, 15); (40, 17). The injection volume, flow rate and column temperature were 3 µL, 0.4 mL/min and 60 °C, respectively.

The MS analysis was performed with ESI-MS/MS operating in positive ionization mode. The next parameters were established: source temperature: 120 °C; nebulization N_2_ flow: 1000 L/h; nebulization N_2_ temperature: 500 °C; cone N_2_ flow: 30 L/h; capillary voltage: 3.2 kv and cone voltage: 30 v. Data obtained were preprocessed using the TargetLynx application manager for MassLynx 4.1 software (Waters Corp., Milford, MA, USA).

Normalization factors were calculated for each lipid (in each sample) by dividing its intensity by the recorded intensity of an appropriate internal standard in the same sample. Any remaining sample injection variable response null values in the corrected data set were replaced with missing values before averaging to form the final data set used for statistical analyses of the study samples [20].

Lipid classification was carried out according to the all-encompassing categorization system put forth by Fahy et al. [21] under the leadership of the International Lipid Classification and Nomenclature Committee (ILCNC) as expressed in the LIPID MAPS initiative (LIPID Metabolites and Pathways Strategy, Cambridge, UK; https://www.lipidmaps.org/; accessed on 28 April 2023.).

### 2.6. Statistics

Statistical analyses were conducted using STATA 15 (College Station, TX, USA) software. Data are expressed as mean and standard error of the mean. The Shapiro–Wilk test was applied to verify the normal distribution of data. Sex-related differences at baseline and postintervention were assessed using a paired Student’s *t*-test. Effects of the 4-week interventions were evaluated using a paired Student’s *t*-test (baseline and postintervention values). Correlation between continuous variables was assessed by means of Pearson’s correlation. All reported *p*-values are two-sided, and a *p*-value of 0.05 or less was considered to indicate statistical significance. To correct for multiple testing (HDL lipid metabolites), false discovery rate *q*-values were calculated using the two-stage, sharpened method described by Benjamini et al. [22]. Sample size was calculated using the JavaScript-based method for sample power and sample size calculation provided in http://www.stat.ubc.ca/~rollin/stats/ssize/n2.html [17]. Based on mean values (pre- and postintervention) of the different lipid species with *p* < 0.05 (Supplementary Table S2) and the pooled standard deviation of both groups, a sample size *n* = 20 gave a study power of >0.80 (type I error = 0.05, two-sided test).

## 3. Results

Twenty subjects (eight men, twelve women) with an average age of 50.6 ± 1.8 years completed both intervention phases and were included in the final analysis. We conducted a double-blind randomized longitudinal crossover exploratory study to investigate the lipidomic changes caused by dietary supplementation with ω3 and PhyS intake on HDLs after four weeks. The demographic and biochemical profiles of the subjects (20) are presented in Table 1.

### 3.1. HDL Lipidome

**Figure 2:**
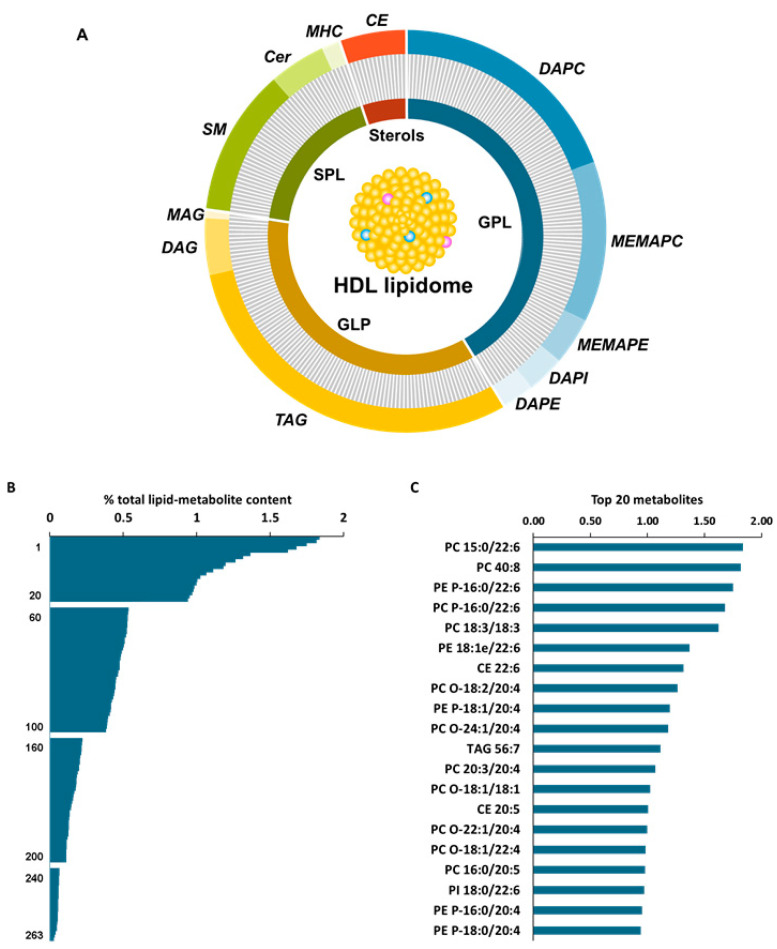
(**A**) Overview of lipid constituents of HDL from healthy population at baseline. (**B**) General map of relative abundance of the 263 detected lipid metabolites. (**C**) Top 20 HDL lipids detected in a healthy population at baseline. CE: cholesteryl ester; Cer: ceramide; DAG: diacylglycerol; DAPC: diacylglycerophosphocholine; DAPE: diacylglycerophosphoethanolamine; DAPI: diacylglycerophosphoinositol; GLP: glycerolipid; GPL: glycerophospholipid; MAG: monoacylglycerol; MEMAPC: 1-ether, 2-acylglycerophosphocholine; MEMAPE: 1-ether, 2-acylglycerophosphoethanolamine; MHC: monohexosylceramide; PE: phosphatidylethanolamine; PI: phosphatidylinositol; SM: sphingomyelin; SPL: sphingolipid and TAG: triacylglycerol.

Analysis of human HDL lipidomic profiles using UPLC-ESI-MS/MS led to the detection of 263 molecular lipid species (Supplementary Tables S1–S4). The detected metabolites were distributed among the four lipid classes, GPLs, GLPs, SPLs and sterols. The most abundant class, representing 42% of the total HDL lipid content, was GPLs, whereas GLP, SPL and sterol content accounted for 35%, 18% and 5%, respectively (Figure 2A). A general map of relative abundance of the 263 lipid metabolites, detected in the HDL at baseline, is shown in Figure 2B.

The identified GPL species belonged to the subclasses of phosphatidylcholine (PC), phosphatidylethanolamine (PE) and phosphatidylinositol (PI). Within the group of PCs are included diacylglycerophosphocholine (DAPC) and 1-ether, 2-acylglycerophosphocholine (MEMAPC), while the PE group includes diacylglycerophosphoethanolamine (DAPE) and 1-ether, 2-acylglycerophosphoethanolamine (MEMAPE) metabolites. The PIs were represented by the diacylglycerophosphoinositol subclass (DAPI). As shown in Figure 2C, the 20 most abundant metabolites (25% of total lipids) belong mostly to the subclasses of DAPC and MEMAPE.

For the second-most abundant lipid class (GLP), triacylglycerols were by far the most numerous lipids (78 compounds); nevertheless, several monoacylglycerols (MAGs) and diacylglycerols (DAGs) were also identified. As shown in Figure 2C, the TAG 56:7 was among the top 20 most abundant HDL metabolites. The SPL group referred to SM, Cer and monohexosylceramides (MHCs), with SM being the best represented (31 lipids). Regarding the CE fraction, 14 metabolites were consistently detected in the HDL, 2 of them located among the top 20 most abundant metabolites (CE 22:6 and CE 20:5).

**Figure 3:**
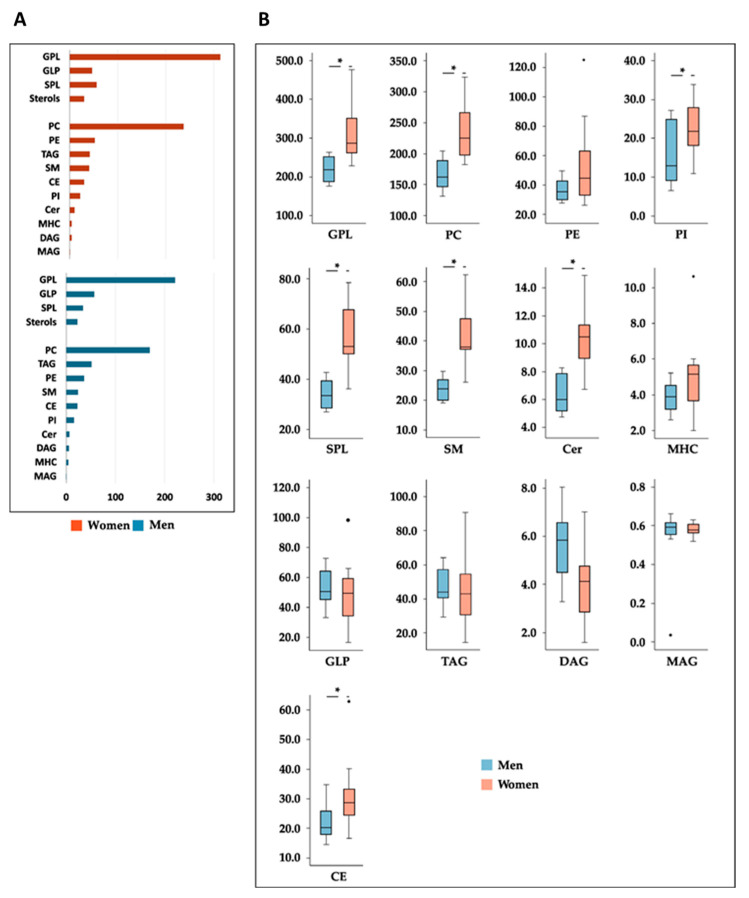
(**A**) Sex disparities in HDL lipidomic signatures at the beginning of the intervention (data given as mean). (**B**) Sex-related differences in HDL composition at baseline (values expressed as median [IQR]). Differences between sexes were analyzed using paired Student’s *t*-test. * Indicates significance (*p* < 0.05). *n* = 12 (women) and *n* = 8 (men). CE: cholesteryl ester; Cer: ceramide; DAG: diacylglycerol; GLP: glycerolipid; GPL: glycerophospholipid; MAG: monoacylglycerol; MHC: monohexosylceramide; PE: phosphatidylethanolamine; PI: phosphatidylinositol; SM: sphingomyelin; SPL: sphingolipid and TAG: triacylglycerol.

The influence of sex on HDL lipid composition, at baseline, in healthy overweight subjects is shown in Figure 3A. Different lipid patterns were observed between both sexes. Higher abundances of GPLs and SPLs were detected in women, while a higher abundance of GLPs was detected in men (*p* < 0.05). According to lipid subclasses, we detected higher significant abundances of PCs, PIs, SM, Cer and CEs (*p* < 0.05) in women than in men (Figure 3B). 

### 3.2. Effects of Dietary Treatments on HDL Lipid Profile

#### 3.2.1. Effect of ω3 Milk on the HDL Lipidome

Comparison between the major lipid classes at the baseline and final intervention (Table 2) revealed that CE classes were significantly increased after intervention compared with the baseline data (31.31 ± 1.70 vs. 27.24 ± 2.43 relative units (RU)/mg HDL protein, respectively; *p* = 0.011), whereas no statistically significant differences were observed for GPLs, GLPs and SPLs. It is also important to mention that PC-DHA (27.97 ± 2.10 to 33.17 ± 1.63 RU/mg HDL protein) and the storage lipids (which include TAGs and CEs, 72.58 ± 3.46 to 85.19 ± 4.70 RU/mg HDL protein) show a statistically significant increase (*p* < 0.05).

**Figure 4:**
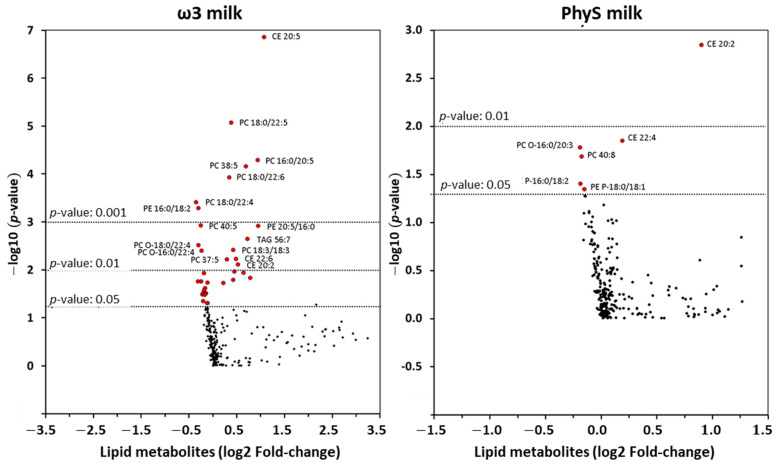
The fold change (log2) between mean concentrations of lipid metabolites in HDL particles according to Student’s *t*-test *p*-values (log10) is shown.

As shown in the “volcano plot” (Figure 4), four weeks of intervention with ω3 milk induced changes in the levels of 35 out of 263 HDL lipid metabolites (*p* < 0.05). Those 35 lipid species included 3 CEs, 5 TAGs and 27 GPLs (12 DAPEs, 10 DAPCs, 4 MEMAPCs and 1 DAPI). The most significantly reduced HDL lipid species after ω3-milk supplementation were PC 18:0/22:4 and PE 16:0/18:2 (*p* < 0.001), while CE 20:5, PC 16:0/20:5, PC 16:0/20:5, PC 38:5 and PC 18:0/22:6 were the metabolites with a more significant increment in HDL after treatment (*p* < 0.001).

**Figure 5:**
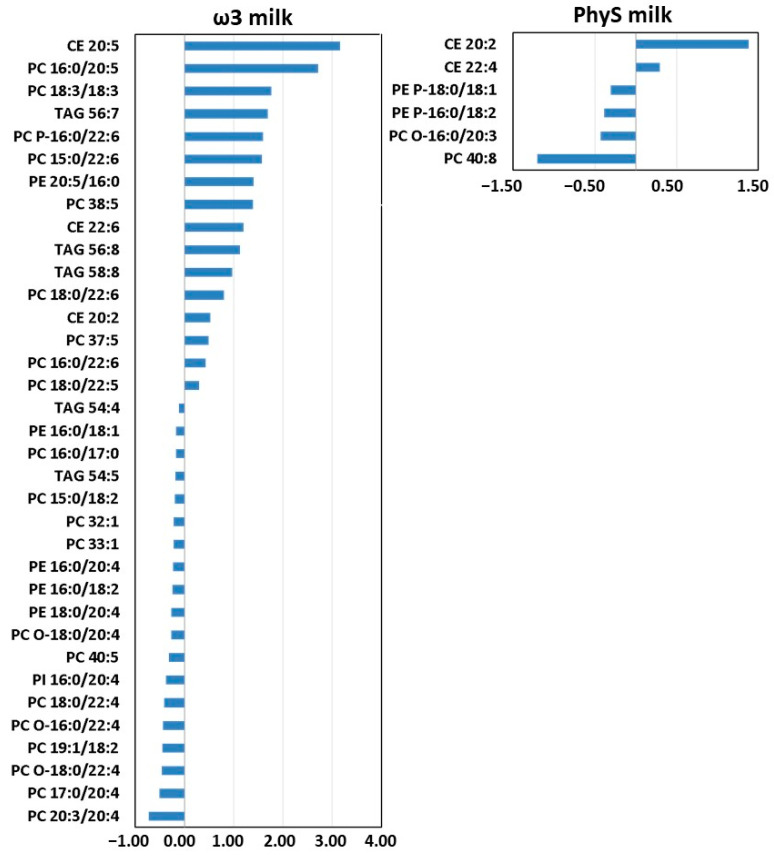
Metabolites with a significant change (*p* < 0.05) after four weeks of ω3 milk or PhyS milk.

Figure 5 shows the changes for all those metabolites that were modified by the intervention. Significant increases were observed for CE 20:2 (+0.52 RU/mg HDL protein), CE 20:5 (+3.16 RU/mg HDL protein) and CE 22:6 (+1.20 RU/mg HDL protein). In fact, at the beginning of the intervention these three compounds represented 39% of the total CEs and at the end of the intervention they represented 50%. FDR adjustment gave *q*-values of 0.037, 0.000 and 0.037 for CE 20:2, CE 20:5 and CE 22:6, respectively. The *q*-value of CE 22:6 indicated that it was the metabolite most affected by the ω3-milk intervention. Concerning the other lipids that were affected by the intervention, although 19 of the compounds decreased, the changes detected for the remaining compounds (13 lipids) were greater than 0.5 RU/mg HDL protein, while the decreases observed for most of the lipids were less than 0.5 RU/mg HDL protein. After FDR adjustment, PC 16:0/20:5, PC 18:0/22:4, PC 18:0/22:5, PC 18:0/22:6 and PC 38:5 gave *q*-values < 0.001. In addition, PC 18:3/18:3, PC 37:5, PC 40:5, PC O-16:0/22:4, PC O-18:0/22:4, PE 16:0/18:2 and PE 20:5/16:0 gave *q*-values < 0.06.

Furthermore, the relationship of distinct enzyme outputs to those of substrates offers an empirical assessment of reaction velocities. Thus, several ratios were calculated in this study to learn the potential enzyme activities related to lipid metabolism (Supplementary Table S5). The ratio of PC with DHA vs. total PC (PC-DHA/PC) has been previously reported as a noninvasive means to detect phosphatidylethanolamine methyltransferase (PEMT) activity, as revised by Cano and Alonso [23]. This ratio was significantly increased after intake of ω3 milk. Additionally, ratios of PC species and PE species, with the same esterified fatty acyl chains, were found to have increased (PC(16:0/18:2)/PE(16:0/18:2), PC(16:0/20:4)/PE(16:0/20:4), PC(16:1e/22:6)/PE(16:1e/22:6), PC(18:0/20:4)/PE(18:0/20:4) and PC(18:2e/20:4)/PE(18:2e/20:4)). Moreover, the TAG-to-DAG ratio was also increased after intake of ω3 milk (fold change 1.28).

**Figure 6:**
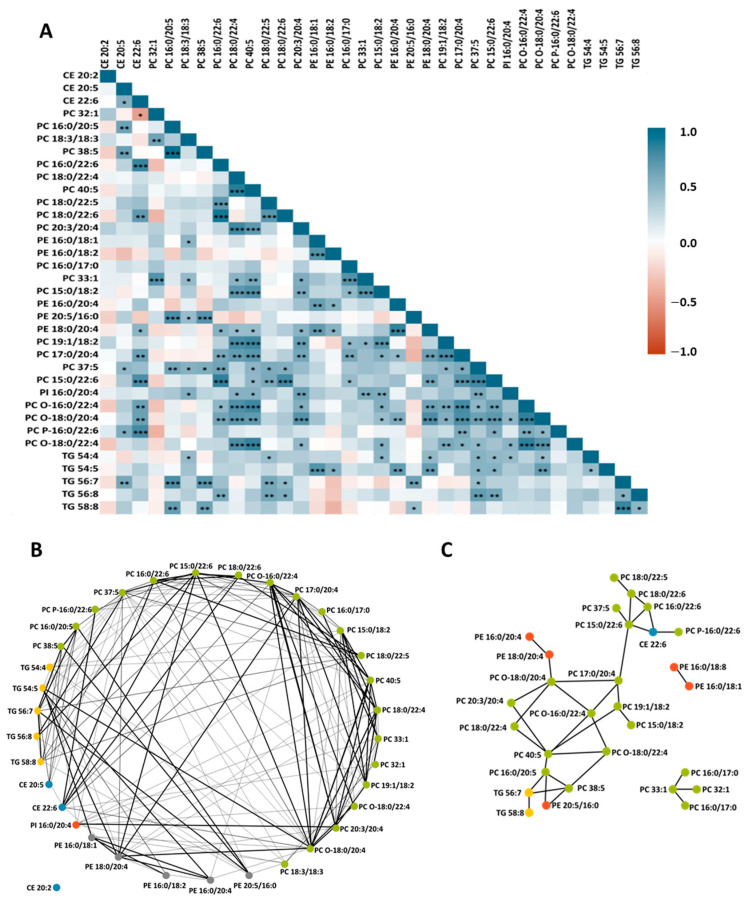
Correlation of differentially expressed lipid metabolites in volunteers who received ω3 milk. (**A**) In this pairwise correlation heat map for those lipids that were significantly different after ω3 supplementation, red shows a negative correlation and blue a positive correlation. Degree of statistically significant correlation is indicated by * (* *p* < 0.05; ** *p* < 0.01; *** *p* < 0.001) (**B**) Correlation networks were constructed based on their Pearson correlation coefficient. In each network, colored nodes refer to different lipid species and lines link correlated pairs. (**C**) Network map with all lipids with strong correlation (rho > 0.7, *p* < 0.01).

To better understand the relationship between the HDL lipids, we correlated the lipid metabolites that showed a significant change after 4 weeks of intervention with the ω3-supplemented milk (Figure 6A). Correlation was observed between all metabolites except for CE 20:2. Correlations were positive except for CE 22:6 vs. PC 32:1 (rho = −0.4945). In addition, correlation networks were constructed based on their Pearson correlation coefficient (Figure 6B), demonstrating that the metabolites were highly correlated with each other, and no cluster was observed when all metabolites were plotted. Given the high relationship between the metabolites, a network map was constructed with all those metabolites that were strongly correlated (rho > 0.7, *p* < 0.01). As shown in Figure 6C, four clusters were identified. Two of them were joined by PC 15:0/22:6 and PC 17:0/20:4 lipids.

#### 3.2.2. Effect of PhyS Milk on the HDL Lipidome

Comparison between the main lipid classes at baseline and at the final intervention (Table 2) revealed that there was no significant change after the intake of PhyS milk (*p* > 0.05). Moreover, no statistically significant changes were observed for other indicators such as PC-DHA, PE-DHA, TAG-SFA and DAG-SFA (*p* > 0.05).

Regarding individual species, only 6 of the 263 metabolites identified were found in different abundances in the HDL particles after PhyS milk supplementation. As shown in Figure 5, CE 20:2 was the most significantly increased HDL lipid species (+1.38 RU/mg HDL protein, *p* < 0.01). In addition to the increase in CE 20:2, four-week intervention with PhyS milk induced a significant increase in CE 22:4 (+ 0.29 RU/mg HDL protein, *p*-value = 0.014). The remaining four compounds showed significant decreases: PC O-16:0/20:3 (−0.01), PC 40:8 (−1.20), PE P-16:0/18:2 (−0.04) and PE P-18:0/18:1 (−0.4) (*p* < 0.05). After FDR adjustment, only CE 20:2 gave a value of 0.014; for the rest of the compounds, the *q*-values were >0.05.

Some changes were found among the ratios calculated to detect preferential fluxes and enzyme potential activities (Supplementary Table S5). Statistically significant increases were observed for PC (16:1e/18:2)/PE (16:1e/18:2), SM/PC and SM + DAG/Cer + PC (*p* = 0.01, 0.00 and 0.01, respectively).

### 3.3. Sex-Related Differences in the Effects of Milk Supplementation

Given that sex-related differences in the HDL lipidome were observed at the beginning of the intervention, this effect was also assessed at the end of the intervention. Higher content was observed for GPLs, PCs, PEs, PIs, SPLs, SM, Cer, MHCs and CEs in women (*p* < 0.05). Men presented a statistically significant higher abundance of GLPs and DAGs (*p* < 0.05) (Figure S1, Supplementary Materials).

## 4. Discussion

It is well known that certain constituents of the diet directly affect the composition and structure of HDL and increase its vasoprotective and anti-inflammatory properties [24]. In this case, lipidomic analysis of HDL has emerged as a useful tool to evaluate the effect of the diet on its composition and to identify new biomarkers of HDL function [11]. Although *n*-3 PUFA (ω3) and PhyS are naturally occurring compounds found in a wide variety of foods, so far little is known about their effect on HDL lipidomes. Here, we performed a double-blind randomized longitudinal crossover exploratory study to search for lipidomic changes induced by dietary supplementation with ω3 and PhyS intake in HDL. Our study revealed three important findings: (i) the HDL lipidome is affected by the supplements in the dietary patterns, (ii) sex-related differences in HDL lipid profiles were detected and (iii) lipids display correlations among various members within the same or different lipid families.

Regarding our first finding, it was recently demonstrated that certain classes of lipids are more sensitive markers of dietary changes (PCs, TAGs and CEs) [12]. A similar behavior was observed after three weeks of dietary intervention with different virgin olive oils in subjects with hypercholesterolemia [11]. In another study, TAGs were modified after a high-fat meal in healthy women [25]. The data supporting the responsiveness of these subclasses to dietary factors are consistent with our observations since the lipids belonging to these subclasses are the ones that were more affected by the type of supplementation. Specifically, CE was the lipid class accounting for major differences between ω3- and PhyS-supplemented milk. Although no significant changes were observed in the GLP class and TAG, DAG and MAG subclasses, the ratio of TAG/DAG was higher after ω3-milk supplementation. This is also relevant because the DAGs have been strongly associated with increased risk of CVD [26]. In addition, it is important to note that ω3-milk intake does not affect the content of TAGs and DAGs esterified with saturated fatty acids. This exploratory lipidomic study, using an LC-ESI-MS/MS strategy, revealed 263 different lipid species from four different lipid classes in HDL. The regular intake of ω3 and PhyS milk resulted in differential HDL lipidome patterns, specific for each dietary intervention. In fact, 13% of the lipids underwent changes in the ω3 group, while only 2% were modified in the PhyS group.

In addition, the effect of the treatment was more evident in various lipids containing ω3 fatty acids. A significant increase in lipids containing DHA (e.g., PC-DHA, PC 15:0/22:6, PC 16:0/22:6, PC 18:0/22:6, TAG 58:8 and CE 22:6) was evidenced after 4 weeks in the ω3 milk group. Additionally, lipids esterified with EPA were also positively affected (e.g., PC 16:0/20:5, PC 38:5, PE 20:5/16:0, TAG 56:8 and CE 20:5). This is directly related to the fact that this group was supplemented with milk containing 150 mg EPA + DHA/100 mL of milk (daily intake of 375 mg). Our observations are also relevant since metabolites such as PC and TAG that contain PUFAs, particularly EPA, DHA and docosapentaenoic acid, showed inverse associations with CVD [26,27,28]. In addition, to date, human lipidomic profiling studies have demonstrated that polyunsaturated CEs show inverse associations with CVD [26,27,28]. This observation is in line with those recently documented in hypertriglyceridemic patients by Peña-de-la-Sancha et al. [14], who demonstrated that DHA and EPA supplementation for 5 weeks induced significant increases (131 and 62%, respectively) in these PUFAs as lipid components in HDL. It is important to note that in the mentioned study, the structural changes in HDL particles were more evident than in our study, but this could be attributed to the doses used (EPA 460 mg and DHA 380 mg, twice a day for 5 weeks) and the different types of patients in the studies.

The alterations in HDL metabolism were further corroborated by the enzymatic activity (related to lipid metabolism). PC is synthesized primarily by the cytidine diphosphate (CDP)-choline pathway or via phosphatidylethanolamine-N-methyltransferase (PEMT) activity [29]. In our study, higher PEMT activity could be attributed to the PC-DHA/PC ratio, due to PEMT preferentially generating PC with long-chain PUFAs, such as DHA [30]. In addition, the PC/PE (with the same fatty acyl chains) ratio also supports higher PEMT activity given that PC is synthesized by the methylation of PE via PEMT activity [31].

The beneficial effect of ω3 intake on HDL particles could also explain the fact that some lipids esterified with arachidonic acid (AA) showed decreases after intervention (PC 17:0/20:4, PC 20:3/20:4, PC O-18:0/20:4, PE 16:0/20:4, PE 18:0/20:4 and PI 16:0/20:4). This is highly relevant since it has previously been proposed that an increase in the EPA-to-AA ratio in HDLs is indicative of a more anti-inflammatory profile of HDL [14]. Supplementation with ω3 milk for 4 weeks does not change the profile of the HDL-SPL or its subclasses (SM, Cer and MHC). These results are in line with those of Zhu et al. [12] who previously proposed that these lipid classes are not ideal indicators of dietary factors since modifications in their concentrations are difficult to achieve following a specific diet. Moreover, the fact that there has not been a significant increase in Cer is relevant because a strong positive association between plasma Cer concentrations and incident CVD risk has been documented [32]. Collectively, these results indicate that regular intake of ω3-enriched milk encourages beneficial alterations in the composition of HDL lipids.

Regarding the second finding, to date, sex-related differences in circulating lipids at molecular lipid species levels have been observed [33,34]; however, to our knowledge little is known about sex-related differences in HDL lipidomes. Recent findings have revealed that the primary disparities between sexes are notably discernible at the subclass level [35]. In our study, sex-related differences in the HDL lipid pattern were already observed at the beginning of the intervention, when we found that HDLs were richer in GPLs, PCs, PIs, SPLs, SM, Cer and CEs. In addition to maintaining the differences observed at the beginning of the intervention, discrepancies were also observed in the content of PEs and MHCs at the end of the intervention. On the contrary, HDLs in men presented higher levels of GLPs and DAGs at the end of the dietary intervention period. Despite the differences observed between sexes at the beginning and at the end of the intervention, CE remained the only class that underwent changes after the intervention with ω3 in both sexes. Two of the conducted studies thus far, with the primary aim of elucidating the effects of dietary supplementation on HDL composition [11,12], have conspicuously omitted an exploration of the potential sex-related influences on alterations in the lipid profile. In another study, only men were included in the study [14]. In addition, gonadal hormones and sex chromosome complement stand out as the main factors affecting lipid metabolism [36]. Therefore, despite the paucity of information on the effect of sex on HDL lipid composition and the limited size of our study, we might speculate that sex hormones account for differences in the HDL lipidome between men and women. Further studies are needed to investigate the potential link between sex hormones and ω3 fatty acid- and PhyS-induced changes in the HDL lipidome.

Together with the fact that the type of supplementation produces profound changes in the HDL lipid profile, our third finding to highlight is that lipid changes are the result of a chain reaction. According to our network, lipid species changes occur in a coordinated fashion involving various members of the same or distinct lipid families. These relationships were previously exposed in an HDL lipidomic study with hypercholesterolemic pigs [37]. In our study, two main clusters were identified that were linked by PC 17:0/20:4 and PC 15:0/22:6. This strong relationship is not surprising, since according to the fatty acid biosynthetic pathway, 20:4 is a precursor of 22:6 [38] and 15:0 is a precursor of 17:0 [39]. Our results highlight the impact of diet on HDLs and that any change in the lipid pattern will lead to a chain reaction affecting potentially all lipid metabolites.

A limitation of our study is the sample size (*n* = 20) of the population. In this regard, the technical characteristics and complexity of high-throughput lipidomics studies are the main reasons for the limited size of these studies. Future studies focusing on differential lipid species should be conducted on larger human samples.

## 5. Conclusions

In summary, we have shown that diet modulates the HDL lipidome. Specifically, four weeks of ω3-milk supplementation produce greater changes in the HDL lipidome than supplementation with PhyS milk. Our results suggest that CE HDL lipids could be useful markers of short-term dietary intake. In addition, PCs, TAGs and CEs esterified with DHA and EPA stand as *n*-3 PUFA (ω3) ingest markers.

Moreover, besides the influence of diet, it is worth noting that the HDL lipidome exhibits sex-dependent variations, as evidenced by distinct patterns observed at different stages of the study. These findings emphasize the importance of considering sex-specific factors when investigating the impact of HDL lipid composition and further highlight the complexity of the interplay between diet and sex in shaping the HDL profile.

These findings are consistent with the results of our prior research on HDL proteomes [15], wherein it was found that the consumption of ω3-enriched milk leads to changes in proteins promoting lipid metabolic processes, a phenomenon not observed with PhyS milk.

In conclusion, these findings strongly support the notion that the impact of diet on the HDL lipid profile is of utmost importance, as it reveals a close relationship among all lipid species. These results underscore the significance of dietary interventions in modulating HDL lipid composition and potentially improving cardiovascular health.

## Figures and Tables

**Table 1:** Obesity-related variables and serum lipid levels before and after 4-week dietary intervention with ω3- or PhyS-supplemented milk.

	ω3 Milk				PhyS Milk			
	Before	After	∆	*p*	Before	After	∆	*p*
Male/female	8/v12	**-**	**-**	**-**	**-**	**-**	**-**	**-**
Age (years)	50.60 ± 1.81	**-**	**-**	**-**	**-**	**-**	**-**	**-**
Weight (kg)	73.77 ± 2.40	73.35 ± 2.39	−0.42	0.061	73.64 ± 2.44	73.32 ± 2.42	−0.32	0.215
BMI (kg/m^2^)	27.04 ± 0.42	26.88 ± 0.42	−0.15	0.062	26.98 ± 0.43	26.87 ± 0.44	−0.11	0.268
WC (cm)								
Females	90.00 ± 1.85	89.42 ± 1.79	0.58	0.538	90.67 ± 1.26	89.33 ± 1.15	1.33	0.092
Males	96.75 ± 2.93	95.88 ± 3.08	0.88	0.111	97.13 ± 2.34	97.13 ± 2.94	0.00	1.000
WHtR (cm/cm)	0.56 ± 0.01	0.56 ± 0.01	−0.01	0.180	0.57 ± 0.01	0.56 ± 0.01	−0.01	0.234
Glucose (mM)	4.76 ± 0.22	4.95 ± 0.26	0.19	0.070	4.74 ± 0.14	4.84 ± 0.27	0.10	0.647
Urea (mM)	6.15 ± 0.28	6.33 ± 0.22	0.18	0.464	5.79 ± 0.20	5.64 ± 0.23	−0.15	0.460
Uric acid (mM)	298.95 ± 17.10	304.45 ± 19.87	5.50	0.494	295.50 ± 18.48	297.30 ± 17.93	1.80	0.752
Creatinine (µM)	67.87 ± 2.68	70.07 ± 2.77	2.20	0.369	66.94 ± 2.21	67.50 ± 2.04	0.56	0.320
Total protein (U/l)	69.46 ± 0.68	68.76 ± 0.83	−0.71	0.257	68.65 ± 0.58	68.33 ± 0.65	−0.32	0.589
GGT (U/l)	30.40 ± 2.35	29.15 ± 4.62	−1.25	0.355	32.05 ± 6.51	28.90 ± 5.06	−3.15	0.078
ALT (U/l)	27.10 ± 3.60	24.90 ± 3.52	−2.20	0.567	23.90 ± 3.08	23.50 ± 2.72	−0.40	0.800
AST (U/l)	22.65 ± 2.03	22.50 ± 2.14	−0.15	0.930	21.65 ± 1.65	21.65 ± 2.00	0.00	1.000
TBARS *	6.60 ± 0.63	6.09 ± 0.49	−0.43	0.325	6.97 ± 0.87	5.04 ± 0.46	−1.64	**0.032**
TAG (mg/dL)	131.44 ± 21.89	102.74 ± 12.49	−28.70	**0.018**	120.37 ± 15.46	122.23 ± 2.45	1.86	0.913
TC (mg/dL)	216.72 ± 8.33	214.24 ± 7.67	−2.48	0.432	218.53 ± 8.10	202.37 ± 7.61	−16.16	**<0.01**
HDLc (mg/dL)	56.22 ± 3.77	55.45 ± 3.60	−0.77	0.521	54.30 ± 4.14	54.19 ± 3.80	−0.12	0.95
NonHDLc (mg/dL)	160.50 ± 8.98	158.80 ± 8.51	−1.70	0.514	164.23 ± 8.45	148.18 ± 8.83	−16.05	0.919
LDLc (mg/dL)	134.55 ± 7.20	138.54 ± 6.96	3.98	0.260	140.45 ± 7.11	124.04 ± 6.90	−16.41	**<0.01**
VLDLc (mg/dL)	26.21 ± 4.36	20.45 ± 2.50	−5.76	**0.017**	23.99 ± 3.08	24.34 ± 4.68	0.35	**<0.01**
TAG/HDLc ratio	2.82 ± 0.61	2.14 ± 0.35	−0.68	**0.037**	2.71 ± 0.48	2.76 ± 0.67	0.67	0.894
TC/HDLc ratio	4.16 ± 0.30	4.16 ± 0.08	−0.01	0.935	4.40 ± 0.32	4.08 ± 0.32	0.32	**0.020**
NonHDLc/HDLc ratio	3.16 ± 0.30	3.16 ± 0.28	−0.01	0.935	3.40 ± 0.32	3.08 ± 0.32	0.32	**0.020**

ALT: alanine transaminase; AST: aspartate transaminase; BMI: body mass index; GGT: gamma-glutamyltransferase; HDLc: high-density lipoprotein cholesterol; LDLc: low-density lipoprotein cholesterol; TAG: triacylglycerol; TC: total cholesterol; VLDLc: very low-density lipoprotein cholesterol; WC: waist circumference; WHtR: Waist-to-height ratio. Data are given as mean ± SEM. Values before and after the 4-week interventions were analyzed using paired Student’s *t*-test. *n* = 20. *p* < 0.05 indicates significance. * Expressed in nmol MDA/mg LDL-protein. Bold is to highlight values statistically significant.

**Table 2:** HDL serum lipid levels before and after 4-week dietary intervention with low-fat milk supplemented with ω3 or PhyS.

Lipids	ω3 Milk			PhyS Milk		
	Before	After	*p*	Before	After	*p*
Total lipids	400.39 ± 21.51	409.34 ± 19.14	0.630	404.74 ± 17.52	385.92 ± 18.60	0.180
GPL	275.37 ± 17.36	272.68 ± 14.84	0.839	277.76 ± 14.46	264.35 ± 13.73	0.416
PC	208.37 ± 11.69	207.91 ± 10.49	0.961	212.11 ± 10.48	204.98 ± 11.29	0.327
DAPC	116.16 ± 5.86	118.69 ± 5.67	0.616	117.95 ± 6.14	114.11 ± 5.75	0.468
MEMAPC	92.21 ± 6.93	89.22 ± 6.32	0.581	94.16 ± 5.56	90.87 ± 6.10	0.343
PE	47.04 ± 5.36	46.81 ± 4.24	0.947	46.12 ± 4.15	41.62 ± 2.72	0.127
MEMAPE	35.60 ± 4.83	34.65 ± 3.68	0.764	35.39 ± 3.96	31.39 ± 2.60	0.155
DAPE	11.44 ± 0.92	12.16 ± 1.11	0.382	10.73 ± 0.86	10.23 ± 0.88	0.306
PI						
DAPI	19.96 ± 1.77	17.96 ± 1.73	0.310	19.53 ± 1.79	17.75 ± 1.30	0.182
GLP	50.61 ± 4.22	59.03 ± 4.92	0.161	51.88 ± 4.16	45.33 ± 3.75	0.118
TAG	45.34 ± 3.93	53.88 ± 4.67	0.140	46.73 ± 3.93	40.41 ± 3.58	0.189
DAG	4.71 ± 0.38	4.60 ± 0.38	0.737	4.58 ± 0.47	4.34 ± 0.34	0.491
MAG	0.56 ± 0.03	0.55 ± 0.03	0.892	0.57 ± 0.01	0.59 ± 0.01	0.145
SPL	47.73 ± 3.52	46.88 ± 3.32	0.725	47.71 ± 3.37	48.17 ± 3.99	0.912
SM	34.33 ± 2.68	33.57 ± 2.45	0.654	33.90 ± 2.45	34.93 ± 2.92	0.472
Cer	8.83 ± 0.66	8.51 ± 0.59	0.590	9.02 ± 0.72	8.51 ± 0.74	0.325
MHC	4.57 ± 0.41	4.79 ± 0.42	0.413	4.79 ± 0.37	4.74 ± 0.51	0.844
Sterols						
CE	27.24 ± 2.43	31.31 ± 1.70	**0.011**	27.95 ± 1.82	28.65 ± 1.93	0.538

CE: cholesteryl ester; Cer: ceramide; DAG: diacylglycerol; DAPC: diacylglycerophosphocholine; DAPE: diacylglycerophosphoethanolamine; DAPI: diacylglycerophosphoinositol; GLP: glycerolipid; GPL: glycerophospholipid; MAG: monoacylglycerols; MEMAPC: 1-ether, 2-acylglycerophosphocholine; MEMAPE: 1-ether, 2-acylglycerophosphoethanolamine; MHC: monohexosylceramide; PE: phosphatidylethanolamine; PI: phosphatidylinositol; SM: sphingomyelin; SPL: sphingolipid and TAG: triacylglycerol. Data are given as mean ± SEM. Values before and after the 4-week interventions were analyzed using paired Student’s *t*-test. *n* = 20. *p* < 0.05 indicates significance. Bold is to highlight values statistically significant.

## Data Availability

The data described in the manuscript, code book and analytic code will be made available upon reasonable request pending scientific approval.

## Supplementary Materials

The following supporting information can be downloaded at https://www.mdpi.com/article/10.3390/nu15163637/s1: Figure S1: Sex-related differences in HDL composition at baseline and after four weeks of ω3-milk intake; Table S1: Level of HDL glycerophospholipids at baseline and after intervention with omega-3- or phytosterol-supplemented milk; Table S2: HDL glycerolipid levels at baseline and after intervention with omega-3 or milk supplemented with phytosterols; Table S3: HDL sphingolipid levels at baseline and after intervention with omega-3 or milk supplemented with phytosterols: Table S4: HDL cholesteryl ester levels at baseline and after intervention with omega-3 or milk supplemented with phytosterols; Table S5: Differences in several ratios after dietary intervention with ω3- or PhyS-supplemented milk.
