# Supplementary material for: Dietary ω3 Fatty Acids and Phytosterols in the Modulation of the HDL Lipidome: A Longitudinal Crossover Clinical Study
Source: Nutrients. 2023 Aug 18;15(16):3637. doi: 10.3390/nu15163637 (PMC10459912; doi:10.3390/nu15163637)
Supplement: Supplementary file 1 [file nutrients-15-03637-s001.zip › nutrients-2565476-supplementary.pdf]

Supplementary Material: Tables and Figures

Dietary ω3 fatty acids and phytosterols in the modulation of the HDL lipidome: a longitudinal crossover clinical study.

Teresa Padro[1,2,‡*], Anallely López-Yerena[1,‡], Antonio Perez[3,4], Gemma Vilahur[1,2], Lina Badimon[1,2,5]

1 Cardiovascular Program-ICCC, Institut d'Investigació Biomèdica Sant Pau (IIB SANT PAU), Barcelona, Spain
2 Centro de Investigación Biomédica en Red Cardiovascular (CIBER-CV), Instituto de Salud Carlos III, Madrid, Spain
3 Servicio de Endocrinología y Nutrición. Hospital de la Santa Creu i Sant Pau, IIB Sant Pau, Universitat Autònoma de Barcelona.
4 CIBER de Diabetes y Enfermedades Metabólicas Asociadas (CIBERDEM), Barcelona, Spain
5 Cardiovascular Research Chair, Universitat Autònoma de Barcelona (UAB), 08193 Barcelona, Spain

‡ These authors contributed equally to this study.

* Corresponding Author

Address for corresponding author (*):

Dr Teresa Padro
Cardiovascular Program-ICCC
Institut d'Investigació Biomèdica Sant Pau (IIB SANT PAU)
Sant Antoni Mª Claret 167, 08025 Barcelona, Spain.
Phone: +34.935565886
E-mail: tpadro@santpau.cat

Table S1. Level of HDL- glycerophospholipids at baseline and after intervention with omega 3- or phytosterol-supplemmented milk.

Lipids	ω3 milk					PhyS milk				
	Before	After	Change	*p*-value	FDR	Before	After	Change	*p*-value	FDR
PC 14:0/14:0	1.66 ± 0.41	0.97 ± 0.15	-0.690	0.128	0.335	1.18 ± 0.21	1.33 ± 0.22	0.156	0.632	0.966
PC 14:0/16:1	2.58 ± 0.47	1.86 ± 0.24	-0.724	0.142	0.343	2.14 ± 0.35	2.23 ± 0.34	0.086	0.860	0.966
PC 14:0/18:2	2.20 ± 0.19	2.00 ± 0.19	-0.200	0.251	0.406	2.11 ± 0.14	2.15 ± 0.20	0.038	0.834	0.966
PC 14:0/20:4	2.96 ± 0.29	2.57 ± 0.25	-0.393	0.128	0.335	2.90 ± 0.21	2.89 ± 0.32	-0.004	0.989	0.997
PC 15:0/18:2	1.54 ± 0.13	1.35 ± 0.11	-0.191	**0.049**	0.200	1.56 ± 0.13	1.50 ± 0.12	-0.064	0.524	0.966
PC 15:0/20:4	2.62 ± 0.23	2.41 ± 0.14	-0.203	0.287	0.445	2.65 ± 0.18	2.60 ± 0.21	-0.056	0.762	0.966
PC 15:0/22:6	7.33 ± 0.88	8.90 ± 0.71	1.569	**0.011**	0.093	7.54 ± 0.79	7.29 ± 0.66	-0.249	0.631	0.966
PC 16:0/16:0	1.07 ± 0.05	1.05 ± 0.05	-0.022	0.629	0.726	1.07 ± 0.07	1.06 ± 0.06	-0.016	0.659	0.966
PC 16:0/17:0	1.15 ± 0.13	0.98 ± 0.10	-0.171	**0.049**	0.200	1.11 ± 0.19	1.01 ± 0.13	-0.094	0.583	0.966
PC 16:0/18:0	1.14 ± 0.06	1.08 ± 0.05	-0.051	0.387	0.553	1.15 ± 0.06	1.09 ± 0.05	-0.058	0.268	0.966
PC 16:0/18:1	1.23 ± 0.05	1.16 ± 0.05	-0.075	0.117	0.330	1.21 ± 0.07	1.21 ± 0.06	0.002	0.981	0.997
PC 16:0/18:2	1.00 ± 0.03	0.98 ± 0.03	-0.021	0.570	0.712	1.00 ± 0.04	1.03 ± 0.04	0.026	0.587	0.966
PC 16:0/19:1	2.26 ± 0.16	2.20 ± 0.12	-0.063	0.456	0.618	2.32 ± 0.16	2.22 ± 0.16	-0.103	0.518	0.966
PC 16:0/20:4	1.37 ± 0.06	1.28 ± 0.05	-0.093	0.180	0.354	1.37 ± 0.05	1.42 ± 0.07	0.053	0.405	0.966
PC 16:0/20:5	3.75 ± 0.36	6.47 ± 0.54	2.719	**0.000**	**0.000**	4.18 ± 0.53	3.23 ± 0.28	-0.950	0.054	0.935
PC 16:0/22:6	3.52 ± 0.16	3.95 ± 0.11	0.434	**0.019**	0.116	3.53 ± 0.15	3.68 ± 0.15	0.148	0.346	0.966
PC 16:1/18:2	2.45 ± 0.31	2.08 ± 0.26	-0.369	0.244	0.406	2.43 ± 0.23	2.62 ± 0.37	0.197	0.620	0.966
PC 17:0/18:1	2.36 ± 0.26	2.13 ± 0.18	-0.228	0.151	0.343	2.32 ± 0.28	2.13 ± 0.22	-0.197	0.384	0.966
PC 17:0/18:2	1.65 ± 0.11	1.61 ± 0.10	-0.033	0.651	0.736	1.73 ± 0.10	1.71 ± 0.10	-0.020	0.774	0.966
PC 17:0/20:3	1.78 ± 0.20	1.67 ± 0.14	-0.110	0.318	0.479	1.89 ± 0.19	1.91 ± 0.19	0.015	0.879	0.966
PC 17:0/20:4	2.50 ± 0.28	1.99 ± 0.16	-0.507	**0.024**	0.139	2.35 ± 0.22	2.49 ± 0.22	0.140	0.345	0.966
PC 17:0/20:4	1.77 ± 0.25	2.29 ± 0.23	0.521	0.156	0.343	2.09 ± 0.24	2.21 ± 0.28	0.116	0.579	0.966
PC 17:1/18:1	2.00 ± 0.22	1.91 ± 0.18	-0.087	0.549	0.702	1.98 ± 0.20	1.90 ± 0.20	-0.080	0.538	0.966
PC 17:1/18:2	2.59 ± 0.23	2.51 ± 0.16	-0.082	0.656	0.736	2.68 ± 0.19	2.65 ± 0.22	-0.029	0.879	0.966
PC 18:0/18:1	1.63 ± 0.08	1.56 ± 0.07	-0.069	0.273	0.429	1.68 ± 0.09	1.62 ± 0.08	-0.055	0.601	0.966
PC 18:0/18:2	1.19 ± 0.04	1.19 ± 0.04	0.005	0.895	0.929	1.22 ± 0.04	1.22 ± 0.04	0.001	0.979	0.997
PC 18:0/20:3	1.57 ± 0.08	1.46 ± 0.08	-0.106	0.172	0.348	1.60 ± 0.09	1.63 ± 0.09	0.021	0.781	0.966
PC 18:0/20:4	1.11 ± 0.07	1.11 ± 0.05	0.005	0.928	0.954	1.12 ± 0.05	1.22 ± 0.06	0.094	0.097	0.935

Lipids	ω3 milk					PhyS milk				
	**Before**	**After**	**Change**	***p*-value**	**FDR**	**Before**	**After**	**Change**	***p*-value**	**FDR**
PC 18:0/22:4	1.87 ± 0.14	1.45 ± 0.13	-0.417	**0.000**	**0.000**	1.89 ± 0.17	1.88 ± 0.19	-0.012	0.941	0.995
PC 18:0/22:5	1.10 ± 0.05	1.40 ± 0.05	0.299	**0.000**	**0.000**	1.18 ± 0.06	1.19 ± 0.06	0.013	0.846	0.966
PC 18:0/22:6	3.48 ± 0.16	4.28 ± 0.16	0.797	**0.000**	**0.000**	3.60 ± 0.19	3.77 ± 0.15	0.172	0.298	0.966
PC 18:1e/20:3	2.62 ± 0.35	2.49 ± 0.27	-0.133	0.629	0.726	2.72 ± 0.26	2.57 ± 0.21	-0.152	0.406	0.966
PC 18:2/18:2	1.83 ± 0.15	1.62 ± 0.13	-0.211	0.172	0.348	2.02 ± 0.17	1.86 ± 0.14	-0.158	0.168	0.935
PC 18:3/18:3	6.54 ± 0.60	8.30 ± 0.77	1.759	**0.004**	**0.040**	6.59 ± 0.47	6.38 ± 0.56	-0.208	0.732	0.966
PC 19:1/18:2	2.26 ± 0.25	1.81 ± 0.16	-0.450	**0.012**	0.094	2.08 ± 0.21	1.99 ± 0.20	-0.092	0.539	0.966
PC 20:0/18:2	2.09 ± 0.15	2.02 ± 0.13	-0.064	0.559	0.707	2.17 ± 0.12	2.00 ± 0.15	-0.167	0.169	0.935
PC 20:0/20:4	2.01 ± 0.09	2.09 ± 0.10	0.077	0.461	0.618	2.04 ± 0.08	2.15 ± 0.11	0.108	0.313	0.966
PC 20:3/20:4	4.15 ± 0.32	3.42 ± 0.26	-0.729	**0.027**	0.149	4.50 ± 0.43	4.06 ± 0.40	-0.441	0.152	0.935
PC 30:0	1.32 ± 0.16	1.08 ± 0.12	-0.242	0.167	0.348	1.20 ± 0.18	1.09 ± 0.12	-0.111	0.599	0.966
PC 31:0	1.14 ± 0.10	1.17 ± 0.11	0.028	0.580	0.717	1.15 ± 0.13	1.13 ± 0.13	-0.021	0.843	0.966
PC 32:1	1.42 ± 0.12	1.20 ± 0.10	-0.220	**0.033**	0.151	1.37 ± 0.15	1.31 ± 0.11	-0.058	0.719	0.966
PC 33:1	2.12 ± 0.14	1.90 ± 0.12	-0.224	**0.018**	0.116	2.14 ± 0.22	2.05 ± 0.19	-0.089	0.708	0.966
PC 34:3	1.27 ± 0.13	1.04 ± 0.15	-0.230	0.174	0.348	1.23 ± 0.13	1.14 ± 0.13	-0.087	0.621	0.966
PC 36:3	1.35 ± 0.05	1.24 ± 0.06	-0.116	0.066	0.234	1.35 ± 0.06	1.35 ± 0.05	0.002	0.967	0.997
PC 37:2	2.79 ± 0.45	2.23 ± 0.19	-0.554	0.203	0.354	2.47 ± 0.24	2.33 ± 0.24	-0.148	0.577	0.966
PC 37:2	2.73 ± 0.26	2.79 ± 0.27	0.065	0.723	0.783	2.97 ± 0.25	2.77 ± 0.25	-0.196	0.158	0.935
PC 37:5	2.83 ± 0.22	3.32 ± 0.20	0.494	**0.006**	**0.055**	2.94 ± 0.22	2.68 ± 0.17	-0.254	0.239	0.966
PC 38:5	1.75 ± 0.07	1.74 ± 0.06	-0.012	0.874	0.916	1.76 ± 0.06	1.76 ± 0.06	0.000	0.997	0.997
PC 38:5	2.35 ± 0.12	3.73 ± 0.30	1.386	**0.000**	**0.000**	2.58 ± 0.22	2.32 ± 0.15	-0.257	0.130	0.935
PC 40:0	1.28 ± 0.07	1.27 ± 0.07	-0.002	0.987	0.987	1.30 ± 0.04	1.28 ± 0.04	-0.014	0.807	0.966
PC 40:5	1.71 ± 0.12	1.39 ± 0.10	-0.313	**0.001**	**0.014**	1.68 ± 0.13	1.69 ± 0.14	0.012	0.896	0.966
PC 40:8	6.81 ± 0.44	7.62 ± 0.52	0.810	0.159	0.343	7.90 ± 0.62	6.70 ± 0.47	-1.204	**0.021**	0.935
PC O-16:0/14:0	1.09 ± 0.10	1.07 ± 0.12	-0.020	0.754	0.805	1.14 ± 0.13	1.11 ± 0.13	-0.029	0.723	0.966
PC O-16:0/16:0	1.47 ± 0.13	1.41 ± 0.13	-0.060	0.364	0.534	1.48 ± 0.13	1.50 ± 0.15	0.020	0.749	0.966
PC O-16:0/18:2	3.00 ± 0.29	2.81 ± 0.37	-0.182	0.432	0.594	3.61 ± 0.39	3.15 ± 0.44	-0.464	0.153	0.935
PC O-16:0/20:3	2.07 ± 0.21	1.88 ± 0.20	-0.187	0.364	0.534	2.23 ± 0.20	1.80 ± 0.11	-0.426	**0.017**	0.935
PC O-16:0/20:4	2.50 ± 0.16	2.21 ± 0.16	-0.291	0.063	0.234	2.59 ± 0.15	2.39 ± 0.13	-0.207	0.124	0.935
PC O-16:0/22:4	2.36 ± 0.21	1.92 ± 0.17	-0.432	**0.004**	**0.040**	2.27 ± 0.16	2.18 ± 0.18	-0.090	0.302	0.966

Lipids	ω3 milk					PhyS milk				
	Before	After	Change	*p*-value	FDR	Before	After	Change	*p*-value	FDR
PC O-18:0/18:2	1.71 ± 0.19	1.58 ± 0.25	-0.125	0.305	0.466	1.92 ± 0.22	1.85 ± 0.31	-0.069	0.751	0.966
PC O-18:0/20:4	2.31 ± 0.17	2.04 ± 0.17	-0.273	**0.031**	0.151	2.33 ± 0.15	2.28 ± 0.17	-0.047	0.614	0.966
PC O-18:0/22:4	2.09 ± 0.25	1.63 ± 0.20	-0.463	**0.003**	**0.037**	2.05 ± 0.20	1.94 ± 0.19	-0.118	0.308	0.966
PC O-18:1/18:1	4.17 ± 0.34	3.99 ± 0.28	-0.171	0.540	0.699	4.14 ± 0.33	4.10 ± 0.33	-0.038	0.847	0.966
PC O-18:1/18:2	2.99 ± 0.22	2.71 ± 0.21	-0.287	0.115	0.330	3.30 ± 0.25	2.98 ± 0.29	-0.326	0.104	0.935
PC O-18:1/22:4	4.01 ± 0.34	3.57 ± 0.28	-0.436	0.103	0.312	3.96 ± 0.25	3.83 ± 0.29	-0.135	0.520	0.966
PC O-18:2/20:4	5.01 ± 0.67	6.18 ± 0.52	1.169	0.089	0.288	5.22 ± 0.55	4.94 ± 0.43	-0.281	0.447	0.966
PC O-20:0/20:4	2.74 ± 0.23	2.50 ± 0.21	-0.235	0.133	0.340	2.72 ± 0.18	2.70 ± 0.20	-0.013	0.893	0.966
PC O-22:0/20:4	3.36 ± 0.28	3.18 ± 0.27	-0.180	0.412	0.578	3.39 ± 0.21	3.30 ± 0.23	-0.081	0.523	0.966
PC O-22:1/20:4	4.01 ± 0.35	3.72 ± 0.30	-0.291	0.245	0.406	4.08 ± 0.27	4.07 ± 0.28	-0.013	0.951	0.996
PC O-24:1/20:4	4.78 ± 0.46	4.53 ± 0.36	-0.243	0.469	0.622	4.80 ± 0.31	4.89 ± 0.36	0.085	0.693	0.966
PC O-34:0	1.39 ± 0.14	1.27 ± 0.13	-0.126	0.105	0.312	1.41 ± 0.12	1.40 ± 0.16	-0.009	0.931	0.994
PC O-38:4	2.98 ± 0.21	2.65 ± 0.17	-0.337	0.066	0.234	3.09 ± 0.18	2.82 ± 0.17	-0.270	0.077	0.935
PC O-38:5	2.80 ± 0.14	2.60 ± 0.14	-0.204	0.188	0.354	2.88 ± 0.13	2.71 ± 0.12	-0.162	0.191	0.966
PC O-40:5	3.07 ± 0.24	2.98 ± 0.21	-0.089	0.607	0.726	3.10 ± 0.18	3.05 ± 0.18	-0.045	0.776	0.966
PC O-42:6	2.92 ± 0.28	3.01 ± 0.33	0.090	0.625	0.726	3.02 ± 0.27	3.08 ± 0.29	0.055	0.704	0.966
PC P-16:0/14:0	1.36 ± 0.12	1.32 ± 0.15	-0.042	0.604	0.726	1.42 ± 0.16	1.32 ± 0.14	-0.094	0.281	0.966
PC P-16:0/16:0	1.55 ± 0.15	1.58 ± 0.13	0.030	0.678	0.753	1.60 ± 0.16	1.57 ± 0.16	-0.030	0.718	0.966
PC P-16:0/18:1	1.98 ± 0.18	1.85 ± 0.13	-0.133	0.251	0.406	1.94 ± 0.16	1.92 ± 0.16	-0.013	0.837	0.966
PC P-16:0/18:2	1.40 ± 0.10	1.37 ± 0.12	-0.037	0.627	0.726	1.45 ± 0.13	1.47 ± 0.13	0.023	0.695	0.966
PC P-16:0/20:3	2.91 ± 0.41	2.56 ± 0.25	-0.354	0.262	0.418	2.74 ± 0.25	2.67 ± 0.25	-0.075	0.637	0.966
PC P-16:0/20:4	2.08 ± 0.16	2.03 ± 0.16	-0.052	0.726	0.783	2.06 ± 0.13	2.10 ± 0.15	0.034	0.737	0.966
PC P-16:0/22:6	6.83 ± 0.76	8.43 ± 0.66	1.600	**0.016**	0.116	6.79 ± 0.53	6.58 ± 0.56	-0.209	0.520	0.966
PC P-17:0/20:4	2.12 ± 0.24	2.08 ± 0.21	-0.040	0.861	0.911	2.17 ± 0.16	2.10 ± 0.16	-0.067	0.522	0.966
PC P-18:0/20:4	2.20 ± 0.21	1.90 ± 0.24	-0.303	0.092	0.289	2.17 ± 0.20	2.15 ± 0.19	-0.024	0.831	0.966
PC P-18:0/20:4	2.19 ± 0.22	2.11 ± 0.21	-0.079	0.689	0.758	2.17 ± 0.17	2.19 ± 0.20	0.021	0.883	0.966
PC P-36:2	2.14 ± 0.21	2.06 ± 0.20	-0.077	0.634	0.726	2.20 ± 0.20	2.16 ± 0.20	-0.042	0.712	0.966
PE 16:0/18:1	0.99 ± 0.08	0.83 ± 0.08	-0.167	**0.045**	0.198	0.90 ± 0.07	0.88 ± 0.08	-0.025	0.778	0.966
PE 16:0/18:2	1.06 ± 0.09	0.82 ± 0.07	-0.246	**0.001**	**0.014**	1.00 ± 0.12	0.93 ± 0.11	-0.078	0.255	0.966
PE 16:0/20:4	1.19 ± 0.10	0.95 ± 0.09	-0.231	**0.018**	0.116	1.02 ± 0.09	1.10 ± 0.11	0.080	0.312	0.966

Lipids	ω3 milk					PhyS milk				
	Before	After	Change	*p*-value	FDR	Before	After	Change	*p*-value	FDR
PE 16:0/22:6	3.82 ± 0.42	4.28 ± 0.49	0.466	0.186	0.354	3.38 ± 0.34	3.34 ± 0.37	-0.042	0.842	0.966
PE 18:0/18:1	1.12 ± 0.15	0.88 ± 0.11	-0.247	0.158	0.343	1.15 ± 0.15	1.10 ± 0.18	-0.052	0.795	0.966
PE 18:0/20:4	1.41 ± 0.13	1.15 ± 0.11	-0.262	**0.033**	0.151	1.26 ± 0.11	1.34 ± 0.14	0.080	0.305	0.966
PE 18:1e/22:6	5.57 ± 1.01	6.72 ± 0.90	1.149	0.068	0.234	5.50 ± 0.84	4.86 ± 0.51	-0.645	0.321	0.966
PE 20:5/16:0	1.85 ± 0.27	3.25 ± 0.41	1.404	**0.001**	**0.014**	2.01 ± 0.31	1.54 ± 0.20	-0.467	0.053	0.935
PE P-16:0/18:2	1.80 ± 0.20	1.55 ± 0.19	-0.250	0.068	0.234	1.99 ± 0.28	1.60 ± 0.18	-0.383	**0.040**	0.935
PE P-16:0/20:4	3.88 ± 0.56	3.11 ± 0.32	-0.769	0.128	0.335	3.84 ± 0.53	3.28 ± 0.29	-0.556	0.170	0.935
PE P-16:0/22:6	7.26 ± 1.01	8.17 ± 0.83	0.912	0.148	0.343	6.91 ± 0.69	6.43 ± 0.50	-0.474	0.384	0.966
PE P-18:0/18:1	2.02 ± 0.25	1.87 ± 0.23	-0.146	0.415	0.578	2.07 ± 0.21	1.76 ± 0.16	-0.308	**0.046**	0.935
PE P-18:0/20:4	3.73 ± 0.49	3.14 ± 0.39	-0.591	0.150	0.343	3.89 ± 0.46	3.51 ± 0.37	-0.380	0.237	0.966
PE P-18:0/22:5 + PE P-20:1/20:4	3.28 ± 0.61	3.30 ± 0.55	0.013	0.977	0.986	3.21 ± 0.46	2.76 ± 0.30	-0.444	0.312	0.966
PE P-18:1/20:4	4.83 ± 0.56	3.96 ± 0.37	-0.873	0.078	0.260	4.86 ± 0.51	4.23 ± 0.33	-0.632	0.112	0.935
PE P-20:0/18:2	1.22 ± 0.14	1.15 ± 0.13	-0.075	0.480	0.629	1.21 ± 0.14	1.16 ± 0.11	-0.054	0.562	0.966
PE P-20:0/20:4	2.00 ± 0.28	1.68 ± 0.22	-0.316	0.150	0.343	1.92 ± 0.24	1.79 ± 0.15	-0.121	0.509	0.966
PI 16:0/18:1	2.86 ± 0.33	2.51 ± 0.25	-0.354	0.201	0.354	2.85 ± 0.33	2.35 ± 0.24	-0.493	0.088	0.935
PI 16:0/18:2	1.75 ± 0.23	1.5 ± 0.22	-0.254	0.199	0.354	1.67 ± 0.25	1.58 ± 0.19	-0.091	0.659	0.966
PI 16:0/20:4	2.18 ± 0.18	1.81 ± 0.18	-0.375	**0.031**	0.151	2.18 ± 0.22	2.00 ± 0.16	-0.185	0.263	0.966
PI 18:0/18:1	2.60 ± 0.32	2.11 ± 0.27	-0.491	0.202	0.354	2.55 ± 0.29	2.06 ± 0.21	-0.498	0.100	0.935
PI 18:0/18:2	1.21 ± 0.16	1.2 ± 0.18	-0.010	0.957	0.975	1.30 ± 0.16	1.22 ± 0.13	-0.081	0.533	0.966
PI 18:0/20:3	2.26 ± 0.24	1.93 ± 0.22	-0.336	0.201	0.354	2.07 ± 0.22	1.99 ± 0.21	-0.075	0.546	0.966
PI 18:0/20:4	3.08 ± 0.27	2.70 ± 0.28	-0.383	0.249	0.406	3.06 ± 0.24	2.87 ± 0.24	-0.196	0.360	0.966
PI 18:0/22:6	4.01 ± 0.41	4.34 ± 0.35	0.329	0.377	0.546	3.85 ± 0.37	3.69 ± 0.29	-0.158	0.565	0.966

Data are given as mean ± SEM. Values before and after the 4-week interventions were analyzed by paired Student's t -test. n = 20. $p < 0.05$ indicates significance.

Table S2. HDL glycerolipid levels at baseline and after intervention with omega-3 or milk supplemented with phytosterols.

Lipids	ω3 milk					PhyS milk				
	Before	After	Change	*p*-value	FDR	Before	After	Change	*p*-value	FDR
TAG 40:0	0.13 ± 0.02	0.13 ± 0.02	0.004	0.834	0.924	0.10 ± 0.01	0.16 ± 0.04	0.064	0.143	0.999
TAG 42:0	0.15 ± 0.03	0.17 ± 0.03	0.020	0.647	0.827	0.12 ± 0.01	0.16 ± 0.03	0.047	0.248	0.999
TAG 42:1	0.18 ± 0.05	0.23 ± 0.05	0.053	0.478	0.656	0.14 ± 0.02	0.19 ± 0.04	0.054	0.287	0.999
TAG 43:1	0.37 ± 0.15	0.60 ± 0.13	0.234	0.287	0.518	0.34 ± 0.05	0.36 ± 0.09	0.027	0.814	0.999
TAG 44:0	0.23 ± 0.05	0.31 ± 0.06	0.082	0.357	0.557	0.21 ± 0.02	0.22 ± 0.04	0.014	0.793	0.999
TAG 44:1	0.31 ± 0.08	0.40 ± 0.08	0.086	0.498	0.674	0.24 ± 0.03	0.29 ± 0.06	0.050	0.504	0.999
TAG 44:2	0.25 ± 0.08	0.35 ± 0.07	0.100	0.369	0.566	0.21 ± 0.03	0.25 ± 0.05	0.037	0.560	0.999
TAG 45:0	0.34 ± 0.12	0.57 ± 0.11	0.228	0.195	0.515	0.35 ± 0.04	0.36 ± 0.08	0.012	0.906	0.999
TAG 45:1	0.38 ± 0.14	0.65 ± 0.14	0.271	0.211	0.515	0.36 ± 0.05	0.39 ± 0.09	0.022	0.850	0.999
TAG 45:2	0.36 ± 0.15	0.61 ± 0.15	0.249	0.269	0.515	0.32 ± 0.04	0.38 ± 0.10	0.051	0.671	0.999
TAG 46:0	0.25 ± 0.06	0.32 ± 0.07	0.063	0.609	0.800	0.23 ± 0.03	0.24 ± 0.04	0.008	0.889	0.999
TAG 46:1	0.31 ± 0.07	0.32 ± 0.05	0.007	0.941	0.988	0.24 ± 0.03	0.26 ± 0.04	0.029	0.618	0.999
TAG 46:2	0.25 ± 0.06	0.32 ± 0.06	0.071	0.447	0.623	0.20 ± 0.02	0.24 ± 0.05	0.037	0.485	0.999
TAG 46:3	0.27 ± 0.07	0.37 ± 0.07	0.101	0.324	0.541	0.23 ± 0.02	0.28 ± 0.06	0.047	0.469	0.999
TAG 46:3	0.27 ± 0.07	0.36 ± 0.07	0.097	0.332	0.541	0.23 ± 0.03	0.27 ± 0.06	0.042	0.509	0.999
TAG 47:0	0.46 ± 0.14	0.68 ± 0.13	0.229	0.249	0.515	0.45 ± 0.05	0.46 ± 0.10	0.006	0.963	0.999
TAG 47:1	0.34 ± 0.11	0.55 ± 0.11	0.203	0.241	0.515	0.33 ± 0.04	0.36 ± 0.08	0.027	0.787	0.999
TAG 48:0	0.20 ± 0.04	0.21 ± 0.03	0.010	0.845	0.925	0.19 ± 0.03	0.19 ± 0.02	0.000	0.996	0.999
TAG 48:1	0.32 ± 0.06	0.29 ± 0.04	-0.028	0.686	0.841	0.26 ± 0.05	0.25 ± 0.03	-0.007	0.906	0.999
TAG 48:2	0.35 ± 0.06	0.33 ± 0.04	-0.018	0.792	0.910	0.30 ± 0.04	0.31 ± 0.04	0.007	0.897	0.999
TAG 48:3	0.30 ± 0.05	0.33 ± 0.05	0.023	0.707	0.845	0.27 ± 0.03	0.31 ± 0.05	0.043	0.421	0.999
TAG 49:1	0.35 ± 0.06	0.43 ± 0.06	0.078	0.413	0.585	0.31 ± 0.03	0.32 ± 0.05	0.007	0.911	0.999
TAG 49:2	0.31 ± 0.07	0.41 ± 0.07	0.105	0.295	0.522	0.29 ± 0.02	0.30 ± 0.05	0.017	0.784	0.999
TAG 49:3	0.39 ± 0.10	0.56 ± 0.09	0.168	0.247	0.515	0.37 ± 0.03	0.39 ± 0.07	0.021	0.796	0.999
TAG 50:0	0.23 ± 0.04	0.22 ± 0.02	-0.007	0.870	0.942	0.21 ± 0.03	0.20 ± 0.02	-0.005	0.907	0.999
TAG 50:1	0.44 ± 0.05	0.36 ± 0.04	-0.078	0.101	0.515	0.38 ± 0.05	0.37 ± 0.03	-0.015	0.782	0.999
TAG 50:2	0.45 ± 0.04	0.39 ± 0.04	-0.066	0.116	0.515	0.41 ± 0.05	0.39 ± 0.03	-0.015	0.775	0.999
TAG 50:3	0.39 ± 0.03	0.36 ± 0.03	-0.032	0.335	0.541	0.37 ± 0.04	0.37 ± 0.04	-0.006	0.855	0.999

Lipids	ω3 milk					PhyS milk				
	Before	After	Change	*p*-value	FDR	Before	After	Change	*p*-value	FDR
TAG 50:4	0.32 ± 0.04	0.29 ± 0.04	-0.033	0.407	0.585	0.33 ± 0.04	0.33 ± 0.05	0.000	0.993	0.999
TAG 51:1	0.34 ± 0.05	0.34 ± 0.04	-0.001	0.992	0.992	0.29 ± 0.03	0.29 ± 0.04	0.000	0.999	0.999
TAG 51:2	0.48 ± 0.05	0.45 ± 0.04	-0.028	0.556	0.741	0.43 ± 0.04	0.42 ± 0.05	-0.005	0.910	0.999
TAG 51:3	0.49 ± 0.05	0.47 ± 0.05	-0.022	0.642	0.827	0.45 ± 0.04	0.46 ± 0.06	0.003	0.931	0.999
TAG 51:4	0.38 ± 0.04	0.40 ± 0.04	0.017	0.680	0.841	0.36 ± 0.03	0.38 ± 0.04	0.018	0.576	0.999
TAG 52:0	0.25 ± 0.03	0.34 ± 0.05	0.092	0.157	0.515	0.23 ± 0.02	0.25 ± 0.03	0.015	0.720	0.999
TAG 52:1	0.38 ± 0.05	0.31 ± 0.04	-0.069	0.176	0.515	0.34 ± 0.05	0.33 ± 0.04	-0.014	0.800	0.999
TAG 52:2	0.67 ± 0.04	0.62 ± 0.04	-0.058	0.076	0.515	0.64 ± 0.05	0.63 ± 0.03	-0.006	0.849	0.999
TAG 52:3	0.48 ± 0.03	0.43 ± 0.03	-0.049	0.094	0.515	0.46 ± 0.04	0.45 ± 0.03	-0.011	0.706	0.999
TAG 52:4	0.35 ± 0.06	0.25 ± 0.06	-0.099	0.139	0.515	0.36 ± 0.11	0.29 ± 0.05	-0.064	0.574	0.999
TAG 52:4	0.43 ± 0.04	0.39 ± 0.04	-0.042	0.276	0.515	0.44 ± 0.04	0.41 ± 0.04	-0.022	0.571	0.999
TAG 52:5	0.40 ± 0.04	0.34 ± 0.04	-0.055	0.236	0.515	0.44 ± 0.06	0.39 ± 0.05	-0.050	0.431	0.999
TAG 53:0	0.42 ± 0.10	0.63 ± 0.11	0.206	0.196	0.515	0.42 ± 0.04	0.42 ± 0.08	-0.003	0.972	0.999
TAG 53:1	0.33 ± 0.07	0.43 ± 0.06	0.104	0.280	0.515	0.30 ± 0.03	0.31 ± 0.05	0.009	0.889	0.999
TAG 53:2	0.62 ± 0.06	0.56 ± 0.04	-0.064	0.192	0.515	0.57 ± 0.05	0.55 ± 0.05	-0.027	0.571	0.999
TAG 53:4	0.51 ± 0.06	0.44 ± 0.05	-0.067	0.253	0.515	0.51 ± 0.05	0.48 ± 0.06	-0.032	0.533	0.999
TAG 53:5	0.50 ± 0.07	0.40 ± 0.05	-0.101	0.148	0.515	0.53 ± 0.09	0.45 ± 0.05	-0.077	0.356	0.999
TAG 54:0	0.31 ± 0.08	0.59 ± 0.13	0.278	0.089	0.515	0.32 ± 0.03	0.32 ± 0.06	0.005	0.950	0.999
TAG 54:1	0.27 ± 0.07	0.61 ± 0.15	0.345	0.054	0.515	0.28 ± 0.04	0.31 ± 0.05	0.034	0.627	0.999
TAG 54:2	0.53 ± 0.05	0.54 ± 0.04	0.003	0.959	0.988	0.53 ± 0.06	0.52 ± 0.06	-0.005	0.888	0.999
TAG 54:3	0.72 ± 0.05	0.64 ± 0.04	-0.076	0.068	0.515	0.73 ± 0.07	0.71 ± 0.06	-0.022	0.578	0.999
TAG 54:4	0.58 ± 0.05	0.47 ± 0.03	-0.109	**0.031**	0.515	0.59 ± 0.06	0.57 ± 0.05	-0.029	0.566	0.999
TAG 54:5	0.46 ± 0.05	0.38 ± 0.03	-0.087	0.129	0.515	0.51 ± 0.07	0.46 ± 0.05	-0.046	0.566	0.999
TAG 54:5	0.90 ± 0.07	0.71 ± 0.08	-0.190	**0.018**	0.414	0.85 ± 0.09	0.80 ± 0.07	-0.046	0.541	0.999
TAG 54:6	0.39 ± 0.07	0.28 ± 0.05	-0.106	0.214	0.515	0.51 ± 0.14	0.39 ± 0.07	-0.119	0.463	0.999
TAG 54:6	0.74 ± 0.05	0.82 ± 0.09	0.076	0.378	0.570	0.74 ± 0.05	0.66 ± 0.06	-0.082	0.171	0.999
TAG 54:7	0.35 ± 0.12	0.18 ± 0.05	-0.164	0.397	0.580	0.74 ± 0.39	0.25 ± 0.08	-0.492	0.234	0.999
TAG 55:1	0.34 ± 0.12	0.61 ± 0.13	0.264	0.178	0.515	0.34 ± 0.04	0.35 ± 0.09	0.010	0.924	0.999
TAG 55:3	0.47 ± 0.05	0.47 ± 0.04	-0.002	0.977	0.988	0.45 ± 0.04	0.41 ± 0.04	-0.037	0.361	0.999
TAG 56:0	0.35 ± 0.11	0.60 ± 0.12	0.245	0.174	0.515	0.36 ± 0.04	0.35 ± 0.08	-0.011	0.916	0.999

Lipids	ω3 milk					PhyS milk				
	Before	After	Change	*p*-value	FDR	Before	After	Change	*p*-value	FDR
TAG 56:1	0.30 ± 0.10	0.59 ± 0.14	0.292	0.122	0.515	0.29 ± 0.04	0.31 ± 0.07	0.022	0.810	0.999
TAG 56:2	0.33 ± 0.06	0.46 ± 0.08	0.133	0.229	0.515	0.34 ± 0.05	0.35 ± 0.06	0.002	0.976	0.999
TAG 56:3	0.46 ± 0.05	0.37 ± 0.03	-0.096	0.116	0.515	0.47 ± 0.08	0.38 ± 0.06	-0.093	0.081	0.999
TAG 56:5	1.60 ± 0.16	1.41 ± 0.13	-0.195	0.320	0.541	1.62 ± 0.18	1.43 ± 0.14	-0.188	0.231	0.999
TAG 56:6	1.11 ± 0.09	1.01 ± 0.09	-0.104	0.303	0.526	1.09 ± 0.10	1.00 ± 0.09	-0.095	0.204	0.999
TAG 56:7	0.92 ± 0.08	0.91 ± 0.09	-0.008	0.942	0.988	0.94 ± 0.09	0.87 ± 0.09	-0.069	0.510	0.999
TAG 56:7	4.47 ± 0.66	6.16 ± 0.81	1.687	**0.002**	0.184	4.54 ± 0.68	3.31 ± 0.35	-1.228	0.066	0.999
TAG 56:8	0.52 ± 0.07	0.63 ± 0.10	0.103	0.384	0.570	0.58 ± 0.12	0.45 ± 0.08	-0.126	0.429	0.999
TAG 56:8	3.54 ± 0.49	4.66 ± 0.55	1.126	**0.015**	0.414	3.83 ± 0.60	2.74 ± 0.29	-1.085	0.094	0.999
TAG 58:1	0.28 ± 0.10	0.50 ± 0.11	0.223	0.160	0.515	0.29 ± 0.04	0.29 ± 0.07	0.004	0.961	0.999
TAG 58:10	1.60 ± 0.20	2.17 ± 0.29	0.576	0.075	0.515	2.04 ± 0.40	1.47 ± 0.20	-0.575	0.235	0.999
TAG 58:2	0.22 ± 0.06	0.37 ± 0.08	0.147	0.155	0.515	0.24 ± 0.03	0.24 ± 0.05	0.006	0.924	0.999
TAG 58:3	0.23 ± 0.05	0.25 ± 0.04	0.017	0.811	0.910	0.25 ± 0.06	0.16 ± 0.03	-0.085	0.180	0.999
TAG 58:6	0.75 ± 0.08	0.72 ± 0.08	-0.029	0.695	0.841	0.75 ± 0.09	0.61 ± 0.06	-0.141	0.111	0.999
TAG 58:8	2.73 ± 0.36	3.70 ± 0.46	0.968	**0.012**	0.414	3.20 ± 0.61	2.12 ± 0.21	-1.080	0.097	0.999
TAG 58:9	2.91 ± 0.37	3.50 ± 0.35	0.587	0.114	0.515	3.48 ± 0.69	2.43 ± 0.26	-1.056	0.169	0.999
TAG 59:1	0.33 ± 0.12	0.53 ± 0.12	0.198	0.258	0.515	0.31 ± 0.04	0.32 ± 0.08	0.017	0.857	0.999
TAG 60:1	0.30 ± 0.10	0.53 ± 0.11	0.230	0.191	0.515	0.31 ± 0.04	0.30 ± 0.07	-0.014	0.881	0.999
TAG 60:2	0.24 ± 0.07	0.39 ± 0.08	0.151	0.175	0.515	0.26 ± 0.03	0.24 ± 0.05	-0.020	0.759	0.999
TAG 60:3	0.22 ± 0.05	0.33 ± 0.06	0.107	0.236	0.515	0.22 ± 0.02	0.21 ± 0.04	0.000	0.993	0.999
DAG 32:0	0.27 ± 0.02	0.30 ± 0.03	0.039	0.278	0.515	0.26 ± 0.02	0.27 ± 0.01	0.008	0.730	0.999
DAG 32:1	0.25 ± 0.04	0.24 ± 0.03	-0.009	0.810	0.910	0.21 ± 0.03	0.20 ± 0.03	-0.009	0.814	0.999
DAG 34:0	0.36 ± 0.03	0.46 ± 0.05	0.103	0.072	0.515	0.36 ± 0.01	0.37 ± 0.02	0.014	0.577	0.999
DAG 34:1	0.47 ± 0.05	0.42 ± 0.06	-0.052	0.274	0.515	0.44 ± 0.07	0.42 ± 0.05	-0.025	0.662	0.999
DAG 34:2	0.21 ± 0.03	0.19 ± 0.02	-0.022	0.247	0.515	0.21 ± 0.03	0.19 ± 0.02	-0.015	0.567	0.999
DAG 36:1	0.75 ± 0.08	0.79 ± 0.09	0.037	0.686	0.841	0.73 ± 0.10	0.71 ± 0.09	-0.018	0.842	0.999
DAG 36:2	0.83 ± 0.08	0.75 ± 0.07	-0.074	0.192	0.515	0.84 ± 0.11	0.80 ± 0.10	-0.041	0.439	0.999
DAG 36:3	0.20 ± 0.03	0.18 ± 0.02	-0.020	0.275	0.515	0.20 ± 0.03	0.18 ± 0.03	-0.015	0.631	0.999
DAG 36:4	0.27 ± 0.03	0.23 ± 0.02	-0.041	0.356	0.557	0.25 ± 0.03	0.24 ± 0.02	-0.013	0.458	0.999
DAG 36:4	0.25 ± 0.03	0.24 ± 0.04	-0.009	0.965	0.988	0.26 ± 0.05	0.21 ± 0.02	-0.054	0.241	0.999

Lipids	ω3 milk					PhyS milk				
	**Before**	**After**	**Change**	***p*-value**	**FDR**	**Before**	**After**	**Change**	***p*-value**	**FDR**
DAG 38:4	0.35 ± 0.02	0.34 ± 0.02	-0.009	0.727	0.857	0.35 ± 0.02	0.32 ± 0.02	-0.027	0.285	0.999
DAG 38:5	0.53 ± 0.04	0.47 ± 0.04	-0.060	0.180	0.515	0.52 ± 0.05	0.47 ± 0.04	-0.046	0.129	0.999
MAG 16:0/0:0/0:0	0.29 ± 0.02	0.29 ± 0.01	-0.006	0.769	0.896	0.30 ± 0.01	0.31 ± 0.01	0.013	0.148	0.999
MAG 18:0/0:0/0:0	0.27 ± 0.01	0.27 ± 0.01	0.001	0.974	0.988	0.27 ± 0.00	0.28 ± 0.00	0.004	0.504	0.999

Data are given as mean ± SEM. Values before and after the 4-week interventions were analyzed by paired Student's t -test. n = 20. $p < 0.05$ indicates significance.

Table S3. HDL sphingolipid levels at baseline and after intervention with omega-3 or milk supplemented with phytosterols.

Lipids	ω3 milk					PhyS milk				
	Before	After	Change	*p*-value	FDR	Before	After	Change	*p*-value	FDR
SM 31:1	0.89 ± 0.10	0.87 ± 0.11	-0.026	0.717	0.981	0.93 ± 0.11	0.93 ± 0.10	-0.002	0.982	0.984
SM 32:1	1.05 ± 0.08	1.04 ± 0.09	-0.001	0.981	0.981	1.08 ± 0.10	1.11 ± 0.10	0.029	0.639	0.984
SM 33:1	1.11 ± 0.12	1.05 ± 0.11	-0.064	0.294	0.981	1.12 ± 0.12	1.16 ± 0.13	0.038	0.551	0.984
SM 36:2	0.95 ± 0.07	0.94 ± 0.06	-0.003	0.938	0.981	0.92 ± 0.06	0.97 ± 0.08	0.053	0.182	0.984
SM 38:0	0.93 ± 0.08	0.90 ± 0.08	-0.032	0.567	0.981	0.87 ± 0.07	0.87 ± 0.07	0.003	0.955	0.984
SM 38:1	0.81 ± 0.04	0.85 ± 0.05	0.040	0.292	0.981	0.81 ± 0.04	0.85 ± 0.05	0.037	0.187	0.984
SM 39:1	1.28 ± 0.11	1.29 ± 0.11	0.014	0.822	0.981	1.30 ± 0.11	1.35 ± 0.12	0.050	0.405	0.984
SM 42:1	1.06 ± 0.08	1.06 ± 0.08	-0.002	0.975	0.981	1.06 ± 0.06	1.05 ± 0.08	-0.004	0.915	0.984
SM 42:3	1.37 ± 0.11	1.35 ± 0.09	-0.020	0.781	0.981	1.34 ± 0.09	1.40 ± 0.11	0.056	0.341	0.984
SM 43:1	1.13 ± 0.15	1.06 ± 0.13	-0.069	0.385	0.981	1.10 ± 0.12	1.12 ± 0.15	0.020	0.833	0.984
SM 43:2	1.75 ± 0.25	1.67 ± 0.18	-0.079	0.593	0.981	1.61 ± 0.17	1.76 ± 0.23	0.150	0.389	0.984
SM d16:1/24:1	2.22 ± 0.18	2.28 ± 0.17	0.064	0.580	0.981	2.21 ± 0.19	2.32 ± 0.20	0.110	0.329	0.984
SM d18:0/14:0	0.84 ± 0.06	0.79 ± 0.07	-0.051	0.367	0.981	0.82 ± 0.08	0.84 ± 0.08	0.014	0.794	0.984
SM d18:0/15:0	0.70 ± 0.07	0.69 ± 0.08	-0.010	0.868	0.981	0.72 ± 0.08	0.72 ± 0.10	0.003	0.968	0.984
SM d18:0/16:0	0.78 ± 0.07	0.73 ± 0.06	-0.052	0.325	0.981	0.77 ± 0.07	0.79 ± 0.08	0.019	0.611	0.984
SM d18:0/18:0	0.84 ± 0.08	0.78 ± 0.07	-0.052	0.412	0.981	0.78 ± 0.08	0.80 ± 0.08	0.021	0.749	0.984
SM d18:0/22:0	0.87 ± 0.08	0.84 ± 0.08	-0.030	0.691	0.981	0.81 ± 0.06	0.81 ± 0.07	-0.004	0.931	0.984
SM d18:1/12:0	0.87 ± 0.08	0.85 ± 0.10	-0.021	0.772	0.981	0.89 ± 0.12	0.89 ± 0.10	-0.001	0.984	0.984
SM d18:1/16:0	0.89 ± 0.06	0.86 ± 0.05	-0.024	0.558	0.981	0.90 ± 0.06	0.91 ± 0.06	0.016	0.641	0.984
SM d18:1/17:0	0.91 ± 0.09	0.93 ± 0.08	0.020	0.653	0.981	0.90 ± 0.08	0.95 ± 0.10	0.051	0.300	0.984
SM d18:1/18:0	0.64 ± 0.04	0.66 ± 0.04	0.015	0.598	0.981	0.64 ± 0.04	0.69 ± 0.05	0.047	0.102	0.984
SM d18:1/22:0	0.88 ± 0.04	0.88 ± 0.05	-0.001	0.975	0.981	0.88 ± 0.04	0.90 ± 0.05	0.021	0.488	0.984
SM d18:1/23:0	1.33 ± 0.10	1.29 ± 0.09	-0.040	0.593	0.981	1.32 ± 0.08	1.34 ± 0.10	0.017	0.750	0.984
SM d18:1/23:1	2.17 ± 0.24	2.10 ± 0.19	-0.072	0.546	0.981	2.10 ± 0.19	2.21 ± 0.22	0.110	0.331	0.984
SM d18:1/24:1 + SM d18:2/24:0	1.43 ± 0.09	1.44 ± 0.08	0.009	0.895	0.981	1.43 ± 0.08	1.43 ± 0.09	0.004	0.939	0.984
SM d18:1/25:0	1.47 ± 0.16	1.41 ± 0.13	-0.059	0.535	0.981	1.45 ± 0.12	1.43 ± 0.16	-0.023	0.787	0.984
SM d18:2/14:0	1.38 ± 0.14	1.27 ± 0.15	-0.115	0.276	0.981	1.36 ± 0.14	1.37 ± 0.15	0.017	0.836	0.984
SM d18:2/16:0	1.40 ± 0.10	1.33 ± 0.10	-0.072	0.400	0.981	1.41 ± 0.10	1.42 ± 0.12	0.008	0.895	0.984

Lipids	ω3 milk					PhyS milk				
	**Before**	**After**	**Change**	***p*-value**	**FDR**	**Before**	**After**	**Change**	***p*-value**	**FDR**
SM d18:2/20:0	0.99 ± 0.08	1.01 ± 0.07	0.024	0.583	0.981	0.97 ± 0.07	1.04 ± 0.09	0.065	0.149	0.984
SM d18:2/22:0	1.05 ± 0.07	1.05 ± 0.08	0.005	0.946	0.981	1.04 ± 0.07	1.09 ± 0.08	0.053	0.205	0.984
SM d18:2/23:0	1.78 ± 0.17	1.73 ± 0.16	-0.046	0.665	0.981	1.77 ± 0.15	1.82 ± 0.17	0.049	0.498	0.984
Cer 40:2	0.95 ± 0.09	0.95 ± 0.09	0.004	0.964	0.981	0.99 ± 0.11	0.93 ± 0.10	-0.058	0.514	0.984
Cer 42:3	1.06 ± 0.10	1.04 ± 0.09	-0.024	0.830	0.981	1.10 ± 0.10	1.03 ± 0.11	-0.074	0.351	0.984
Cer d18:1/16:0	0.52 ± 0.03	0.57 ± 0.04	0.052	0.135	0.981	0.54 ± 0.04	0.52 ± 0.04	-0.015	0.431	0.984
Cer d18:1/18:0	0.92 ± 0.06	0.90 ± 0.05	-0.025	0.693	0.981	0.94 ± 0.08	0.94 ± 0.08	-0.004	0.932	0.984
Cer d18:1/20:0	0.96 ± 0.08	0.92 ± 0.07	-0.044	0.497	0.981	0.97 ± 0.08	0.90 ± 0.09	-0.070	0.295	0.984
Cer d18:1/21:0	1.00 ± 0.09	0.96 ± 0.10	-0.035	0.550	0.981	1.03 ± 0.10	0.95 ± 0.09	-0.080	0.332	0.984
Cer d18:1/22:0	0.61 ± 0.05	0.56 ± 0.04	-0.048	0.381	0.981	0.63 ± 0.05	0.58 ± 0.06	-0.053	0.270	0.984
Cer d18:1/23:0	0.40 ± 0.03	0.37 ± 0.03	-0.026	0.423	0.981	0.42 ± 0.04	0.39 ± 0.04	-0.030	0.374	0.984
Cer d18:1/24:0	0.45 ± 0.04	0.41 ± 0.03	-0.043	0.351	0.981	0.45 ± 0.04	0.42 ± 0.04	-0.035	0.331	0.984
Cer d18:1/24:1+Cer d18:2/24:0	1.03 ± 0.09	0.95 ± 0.07	-0.076	0.356	0.981	1.02 ± 0.08	0.98 ± 0.10	-0.042	0.533	0.984
Cer d18:1/25:0	0.53 ± 0.05	0.53 ± 0.04	0.004	0.938	0.981	0.54 ± 0.06	0.51 ± 0.06	-0.032	0.597	0.984
Cer d43:1	0.41 ± 0.05	0.35 ± 0.03	-0.061	0.086	0.981	0.39 ± 0.04	0.37 ± 0.04	-0.020	0.594	0.984
MHC d18:1/16:0	0.66 ± 0.05	0.74 ± 0.06	0.075	0.100	0.981	0.69 ± 0.05	0.70 ± 0.04	0.009	0.804	0.984
MHC d18:1/22:0	1.36 ± 0.14	1.45 ± 0.14	0.090	0.445	0.981	1.42 ± 0.11	1.43 ± 0.17	0.013	0.899	0.984
MHC d18:1/23:0	1.42 ± 0.12	1.44 ± 0.14	0.015	0.865	0.981	1.50 ± 0.13	1.47 ± 0.18	-0.028	0.808	0.984
MHC d18:1/24:0	1.12 ± 0.11	1.16 ± 0.12	0.047	0.548	0.981	1.19 ± 0.10	1.14 ± 0.13	-0.048	0.522	0.984

Data are given as mean ± SEM. Values before and after the 4-week interventions were analyzed by paired Student's t -test. n = 20. $p < 0.05$ indicates significance.

Table S4. HDL- cholesteryl ester levels at baseline and after intervention with omega-3 or milk supplemented with phytosterols.

Lipids	ω3 milk					PhyS milk				
	Before	After	Change	*p*-value	FDR	Before	After	Change	*p*-value	FDR
CE 16:0	1.08 ± 0.05	1.11 ± 0.05	0.025	0.519	0.727	1.10 ± 0.05	1.09 ± 0.05	-0.007	0.815	0.891
CE 16:1	1.07 ± 0.12	0.90 ± 0.07	-0.170	0.087	0.203	1.08 ± 0.12	1.00 ± 0.11	-0.082	0.472	0.891
CE 17:0	1.42 ± 0.20	1.41 ± 0.14	-0.006	0.960	0.960	1.45 ± 0.18	1.42 ± 0.16	-0.030	0.800	0.891
CE 17:1	1.58 ± 0.19	1.48 ± 0.12	-0.098	0.513	0.727	1.61 ± 0.20	1.45 ± 0.14	-0.155	0.346	0.807
CE 18:0	1.05 ± 0.08	1.08 ± 0.07	0.024	0.637	0.811	1.10 ± 0.07	0.98 ± 0.06	-0.114	0.080	0.373
CE 18:1	1.45 ± 0.08	1.45 ± 0.06	-0.005	0.936	0.960	1.45 ± 0.07	1.44 ± 0.08	-0.010	0.864	0.891
CE 18:2	1.02 ± 0.05	1.08 ± 0.05	0.055	0.109	0.218	1.05 ± 0.06	1.07 ± 0.06	0.019	0.552	0.891
CE 18:3	1.78 ± 0.20	1.61 ± 0.16	-0.173	0.068	0.190	1.84 ± 0.19	1.78 ± 0.17	-0.058	0.712	0.891
CE 20:2	1.66 ± 0.26	2.19 ± 0.31	0.525	**0.008**	**0.037**	1.92 ± 0.31	3.30 ± 0.54	1.385	**0.001**	**0.014**
CE 20:3	2.24 ± 0.29	2.06 ± 0.13	-0.184	0.421	0.727	2.19 ± 0.16	2.21 ± 0.15	0.018	0.837	0.891
CE 20:4	1.50 ± 0.10	1.47 ± 0.07	-0.028	0.747	0.872	1.51 ± 0.06	1.56 ± 0.08	0.056	0.241	0.675
CE 20:5	3.92 ± 0.48	7.08 ± 0.50	3.164	**0.000**	**0.000**	4.23 ± 0.48	3.65 ± 0.32	-0.578	0.175	0.612
CE 22:4	2.14 ± 0.21	1.89 ± 0.15	-0.254	0.061	0.190	2.08 ± 0.13	2.37 ± 0.18	0.295	**0.014**	0.098
CE 22:6	5.31 ± 0.51	6.51 ± 0.32	1.195	**0.006**	**0.037**	5.35 ± 0.36	5.31 ± 0.36	-0.043	0.891	0.891

Data are given as mean ± SEM. Values before and after the 4-week interventions were analyzed by paired Student's t -test. n = 20. $p < 0.05$ indicates significance.

Table S5. Differences in several ratios after dietary intervention with ω3- or PhyS- supplemented milk.

Ratios	ω3 milk				PhyS milk			
	Before	After	*p*-value	Fold change	Before	After	*p*-value	Fold change
PC(16:0/18:1) / PE(16:0/18:1)	1.44 ± 0.18	1.62 ± 0.15	0.13	1.21	1.50 ± 0.14	1.67 ± 0.21	0.26	1.13
PC(16:0/18:2) / PE(16:0/18:2)	1.14 ± 0.15	1.42 ± 0.16	**0.03**	1.33	1.22 ± 0.13	1.34 ± 0.14	0.19	1.16
PC(16:0/20:4) / PE(16:0/20:4)	1.42 ± 0.24	1.72 ± 0.26	**0.03**	1.23	1.57 ± 0.18	1.70 ± 0.30	0.39	1.03
PC(16:0/22:6) / PE(16:0/22:6)	1.24 ± 0.25	1.26 ± 0.19	0.88	1.08	1.38 ± 0.24	1.52 ± 0.28	0.08	1.09
PC(16:1e/18:2) / PE(16:1e/18:2)	0.88 ± 0.07	1.01 ± 0.08	0.11	1.19	0.85 ± 0.06	1.01 ± 0.08	**0.01**	1.26
PC(16:1e/20:4) / PE(16:1e/20:4)	0.62 ± 0.05	0.74 ± 0.06	0.07	1.27	0.62 ± 0.04	0.68 ± 0.04	0.22	1.16
PC(16:1e/22:6) / PE(16:1e/22:6)	1.01 ± 0.07	1.13 ± 0.09	**0.03**	1.11	1.04 ± 0.07	1.06 ± 0.07	0.74	1.06
PC(18:0/20:4) / PE(18:0/20:4)	0.93 ± 0.14	1.21 ± 0.17	**0.00**	1.31	1.03 ± 0.12	1.09 ± 0.13	0.39	1.06
PC(18:1e/18:1) / PE(18:1e/18:1)	2.35 ± 0.20	2.62 ± 0.26	0.37	1.21	2.17 ± 0.17	2.63 ± 0.31	0.13	1.25
PC(18:2e/20:4) / PE(18:2e/20:4)	1.10 ± 0.09	1.73 ± 0.15	**0.00**	1.69	1.19 ± 0.12	1.23 ± 0.09	0.73	1.18
PC-DHA / PC	0.13 ± 0.00	0.16 ± 0.00	**0.00**	1.23	0.14 ± 0.00	0.14 ± 0.00	0.87	1.01
SM / PC	0.16 ± 0.01	0.16 ± 0.01	0.35	0.99	0.16 ± 0.01	0.17 ± 0.01	**0.00**	1.07
SM + DAG / Cer + PC	0.18 ± 0.01	0.18 ± 0.00	0.19	0.98	0.17 ± 0.01	0.18 ± 0.01	**0.01**	1.06
TAG / DAG	9.83 ± 0.58	12.15 ± 0.82	**0.01**	1.28	11.14 ± 1.08	9.64 ± 0.72	0.23	0.98

Values are given as mean ± SEM. Values before and after the 4-week interventions were analyzed by paired Student's t -test. n = 20. $p < 0.05$ indicates significance.

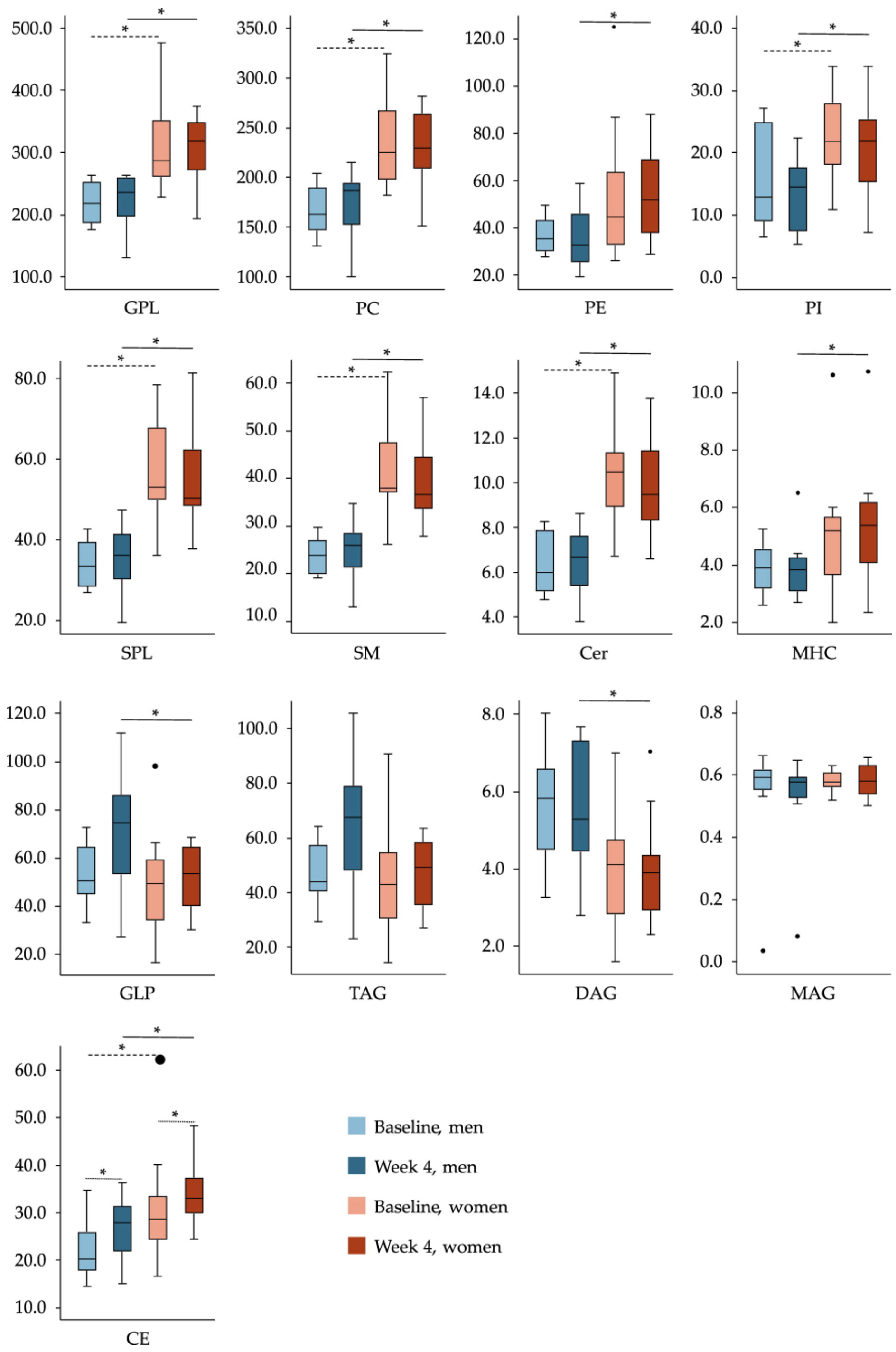

Figure S1. Sex-related differences in HDL composition at baseline and after four-week of ω3-milk intake. Values expressed as median [IQR]). Dashed lines signify comparison between sexes at baseline. Black line means comparison between sexes at the end of the intervention. Dotted line means comparison between sexes between the beginning and the end of the intervention. *Indicates significance ($p < 0.05$). n = 12 (women) and n = 8 (men). CE: Cholesteryl esters; Cer: Ceramides; DAG: Diacylglycerols; GLP: glycerolipids; GPL: glycerophospholipids; MAG: Monoacylglycerols; MHC: Monohexosylceramides; PE: Phosphatidylethanolamines; PI: Phosphatidylinositols; SM: Sphingomyelin; SPL: sphingolipids and TAG: Triacylglycerols.
